# YAP1-CPNE3 positive feedback pathway promotes gastric cancer cell progression

**DOI:** 10.1007/s00018-024-05178-3

**Published:** 2024-03-17

**Authors:** Xuan Li, Hongguang Zhong, Qianqian Shi, Ruiwen Ruan, Chunye Huang, Qin Wen, Shaocheng Zeng, Yang Xia, Qinru Zeng, Jianping Xiong, Shanshan Wang, Jun Chen, Wan Lei, Jun Deng

**Affiliations:** 1 Department of Oncology, The First Affiliated Hospital, Jiangxi Medical College, Nanchang University, Nanchang, Jiangxi People’s Republic of China; 2 Jiangxi Key Laboratory for Individual Cancer Therapy, Nanchang, Jiangxi People’s Republic of China; 3 Department of Pathology, The First Affiliated Hospital, Jiangxi Medical College, Nanchang University, Nanchang, Jiangxi People’s Republic of China; 4 Postdoctoral Innovation Practice Base, The First Affiliated Hospital of Nanchang University, Nanchang, 330006 People’s Republic of China

**Keywords:** Gastric cancer, CPNE3, YAP1, TEADs

## Abstract

Hippo-Yes-associated protein 1 (YAP1) plays an important role in gastric cancer (GC) progression; however, its regulatory network remains unclear. In this study, we identified Copine III (*CPNE3*) was identified as a novel direct target gene regulated by the YAP1/TEADs transcription factor complex. The downregulation of CPNE3 inhibited proliferation and invasion, and increased the chemosensitivity of GC cells, whereas the overexpression of CPNE3 had the opposite biological effects. Mechanistically, CPNE3 binds to the YAP1 protein in the cytoplasm, inhibiting YAP1 ubiquitination and degradation mediated by the E3 ubiquitination ligase β-transducin repeat-containing protein (β-TRCP). Thereby activating the transcription of YAP1 downstream target genes, which creates a positive feedback cycle to facilitate GC progression. Immunohistochemical analysis demonstrated significant upregulation of CPNE3 in GC tissues. Survival and Cox regression analyses indicated that high CPNE3 expression was an independent prognostic marker for GC. This study elucidated the pivotal involvement of an aberrantly activated CPNE3/YAP1 positive feedback loop in the malignant progression of GC, thereby uncovering novel prognostic factors and therapeutic targets in GC.

**Supplementary Information:**

The online version contains supplementary material available at 10.1007/s00018-024-05178-3.

## Introduction

Gastric cancer (GC) is estimated to cause 670,000 new cases and 490,000 deaths in China [1]. Although surgery and chemotherapy may extend a patient's lifespan, the 5-year survival rate of patients with GC is typically less than 35% [2–4]. Therefore, it is important to identify novel prognostic markers and therapeutic strategies for GC.

The Hippo-Yes-associated protein 1 (YAP1) pathway is involved in GC [5–7]. Intestinal Laurence-grade GC exhibited the highest levels of YAP1 expression [8]. The rate of YAP1 protein expression in GC is approximately 68.7%, and YAP1 overexpression has been correlated with progression, metastasis, and poor prognosis in patients with GC [9, 10]. Our group has previously studied the Hippo-YAP1 pathway and discovered that the USP49-YAP1 feedback loop promotes GC progression [11]. Drugs that currently inhibit the YAP1 protein in GC include verteporfin, metformin, AICAR, and TED-347 [11–14]; however, relatively few studies have been conducted on the potential uses of YAP1-specific inhibitors in cancer. Therefore, understanding the YAP1 regulatory network in GC is crucial.

Copine III (CPNE3) is a Ca2-dependent phospholipid-binding protein belonging to the copine (*CPNE*) gene family. CPNE3 has one structural domain and two C2Ds (C2D-A and C2D-B) responsible for protein binding [15, 16]. Although CPNE3 has not been extensively studied, it is known to play an important role in various cancerous processes. A previous study found that the *CPNE3* gene may promote breast cancer by interacting with phosphorylated erb-b2 receptor tyrosine kinase 2 [17]. Colorectal cancer patients with lower levels of exosomal CPNE3 have better disease-free survival and overall survival (OS), suggesting that CPNE3 can serve as a diagnostic and prognostic biomarker [18]. CPNE3 may promote the migration and invasion of non-small-cell lung cancer through the activation of focal adhesion kinase signaling and interaction with receptor for activated C kinase 1 [19]. However, no studies have investigated the function or clinical significance of CPNE3 in GC.

In this study, we explored *CPNE3* as a novel downstream target of the YAP1/ TEADs transcription factor complex, and investigated the mechanism by which CPNE3 inhibits YAP1 ubiquitination by competitively binding to YAP1 with β-transducin repeat-containing protein (β-TRCP), which promotes proliferation, invasion, and chemoresistance in GC cells. In conclusion, we intend to provide new insights into the role of the CPNE3-YAP1 axis in GC development and assess whether it should be investigated further as a predictive biomarker for GC and as a target for GC therapy.

## Materials and methods

### Patient sample and clinical data collection

Eight fresh GC and matched non-cancerous mucosal tissue samples were collected from the operation theater and promptly snap-frozen in liquid nitrogen and preserved at 80 °C until use. Patient information for the eight cases of fresh GC tissues is shown in Supplementary Table S1. Additionally, 288 paraffin-embedded GC tissue samples were collected from the Department of Pathology at the First Affiliated Hospital of Nanchang University. Samples were collected from patients with GC who were admitted to our hospital between March 2016 and March 2019. All patients provided written informed consent to participate in the study. This study was approved by the Ethics Committee of the First Affiliated Hospital of Nanchang University [Ethical No. (2023) CDYFYYLK (04-020)].

### Cell culture

The immortalized gastric epithelial cell line GES-1 and human GC cell lines AGS, BGC-823, HGC-27, MKN-28, and MKN-45 were acquired from the Shanghai Institute of Cell Biology, China Academy of Sciences. MKN-45 and BGC-823 human GC cell lines were grown in Roswell Park Memorial Institute 1640 medium (RPMI, HyClone, Logan, UT, USA) supplemented with 10% fetal bovine serum (FBS). HEK-293 T, MKN-28, HGC-27, and AGS cells were cultured in Dulbecco's modified Eagle's medium (DMEM, Invitrogen, Carlsbad, CA, USA) supplemented with 10% FBS. All cells were cultured at a steady temperature of 37 °C and 5% CO_2_ in a humidified incubator [20].

### Protein extraction and western blotting (WB)

Total proteins were extracted using RIPA buffer with a protease inhibitor cocktail at 4 °C at 48 h post-transfection. Subsequently, proteins were isolated and transferred onto a NC membrane (PALL, cat. no. 66485). Specific experimental steps were performed as described previously [11]. The primary antibodies used are listed in Supplementary Table S2.

### RNA extraction, quantitative reverse transcription polymerase chain reaction (RT-qPCR) assay, and transcriptome sequencing

Total RNA was isolated from the cell samples for RT-qPCR using the Invitrogen RNA-easy Isolation Reagent. Supplementary Table S3 provides an overview of the primers used for RT-qPCR to identify the mRNAs. Specific experimental steps were conducted as described previously [11]. Each sample was tested three times, and the internal control gene *GAPDH* was used to normalize the PCR results.

### RNA interference, lentivirus, plasmid construction, and transfection

GenePharma (Shanghai, China) designed and synthesized three distinct small interference RNA (siRNAs) and short hairpin RNA (shRNAs) targeting CPNE3, which were transiently transfected into BGC-823 cells using Lipofectamine 2000 (Thermo Fisher Scientific, cat. no. 11668027) according to the manufacturer's instructions. Puromycin-resistant clones were generated by transfecting BGC-823 cells with the predesigned shRNAs. Sequencing information for the siRNAs, plasmid, shRNAs, and lentiviruses is provided in Supplementary Table S4.

### Cell proliferation, colony formation, and chemotherapy sensitivity assays

Proliferation and chemoresistance of GC cells were assessed using Cell Counting Kit-8 (CCK-8) (GLPBIO, cat. no. GK10001) by subjecting the transfected cells to gradient doses of 5-fluorouracil (CSNpharm, cat. no. CSN19496), or docetaxel (CSNpharm, cat. no. CSN12495) [11]. These experiments were conducted as described previously [21]. All tests were performed in triplicates, at least.

### Migration and invasion assays

Transfected cells were cultivated in transwell chambers with 200 μL of serum-free RPMI 1640 or DMEM medium. The bottom compartment was supplemented with medium containing 20% FBS as a chemoattractant. Notably, in the invasion experiment, the transwell chamber was cleaned and hydrated with BD adhesive before cell seeding. After 24 h, cells were fixed in methanol and stained with crystal violet. Three independent experiments were performed under identical conditions.

### Immunofluorescence

Cells were seeded in 6-well plates for 24 h after transient transfection with siCPNE3 or HA-CPNE3 plasmids for 48 h, along with their corresponding negative controls. The experiments were conducted as described previously [22]. Finally, cells were observed under a fluorescence microscope (Leica). Three independent experiments were performed under identical conditions.

### Immunohistochemistry analysis (IHC)

IHC was performed with a normal inverted microscope (NION, Japan) by using an anti-YAP1 antibody (1:200; Cell Signaling Technology), anti-CPNE3 antibody (1:50; abcam, Proteintech), anti-cysteine-rich angiogenic inducer 61 (CYR61) antibody (1:100; Proteintech), and anti-RAD51 antibody (1:100; Proteintech). IHC was performed as described previously [11]. Two pathologists assessed the staining results based on the proportion of positively stained cells and the staining strength.

### Dual-luciferase reporter gene assay

A total of six groups of experiments were conducted. To identify changes in *CPNE3* promoter activity, HEK-239 T cells were co-transfected with a GPL4-CPNE3 luciferase reporter plasmid, YAP1, YAP1 + TEAD1/TEAD2/TEAD3/TEAD4, or vehicle plasmids. Cells were lysed 48 h after transfection and dual-luciferase activity was tested using the Dual-Luciferase Kit (YESEN, cat. no. 11402ES60) [11].

### Generation of stable cells and establishment of cell line-derived xenograft (CDX) in mice

BGC-823 cells were infected with a lentivirus encoding a single guide RNA (sgRNA) targeting CPNE3 (sgRNA-CPNE3) or negative control (sgRNA-NC) for 96 h. According to our preceding study, transfected cells were exposed to puromycin (Solarbio, cat P8230, 2 µg/ml) for the construction of stable knockdown cells. Four-week-old female BALB/c nude mice were randomly divided into two groups (n = 10 per group) and subcutaneously injected with 2 × 10^6^ BGC-823 cells that stably expressed lentiviruses encoding sgRNA-CPNE3 or sgRNA-NC. Four weeks after injection, the mice were sacrificed under anesthesia and tumor samples were collected for similar analyses.

### Establishing patient-derived xenograft (PDX) in mice and treatment of lentivirus

Human GC tumor samples (F0 tumors) were surgically removed and subcutaneously injected into immunodeficient mice to induce PDX growth. When tumor growth reached 800 mm^3^ in the developed primary tumor models (P0), they were sliced, chopped into 3 × 3 × 3 mm pieces, and subcutaneously re-engrafted in 4-week female BALB/c nude mice (P1). When the xenograft tumor volume reached approximately 100 mm^3^, the mice were divided into control and treatment groups of ten animals each. The control and treatment group mice received intratumoral 50 µL doses of sgRNA-CPNE3 or sgRNA-NC. The specific experimental were conducted according to previously experiments [11].

### Molecular docking

First, we obtained the three-dimensional (3D) structure of YAP1 from the Protein Data Bank (PDB) database and the forecast-predicted the 3D structure model of CPNE3 from the Swiss-Model Repository database to confirm the link between CPNE3 and YAP1. The findings of our subsequent molecular docking study using ZDOCK for the 3D structures of CPNE3 and YAP1 revealed that CPNE3 can assemble a reliable protein complex with YAP1.

### Co-immunoprecipitation (Co-IP) assay

According to the instructions of the BeaverBeads™ Protein A/G Immunoprecipitation Kit (22202-100) for exogenous Co-IP, cell lysates were incubated with 8 μL of anti-Flag or anti-HA beads. For endogenous Co-IP, cell lysates were immunoprecipitated with anti-CPNE3 or anti-YAP1 polyclonal antibodies, and specific experiments were conducted in accordance with previous studies [11].

### Chromatin immunoprecipitation (ChIP)

ChIP assays were performed according to the manufacturer's instructions using a ChIP Assay Kit (Beyotime, cat. no. 2078). Using Flag-YAP1 and TEADs plasmids transfection, ChIP was performed in BGC-823 cells. The cells were lysed by ultrasonication (SONICS VCX750, USA) and the supernatant was treated with magnetic beads coupled with either a normal rabbit IgG antibody or an anti-YAP1 primary antibody (Cell Signaling Technology, cat. no. D8H1X) in an immunoprecipitation buffer overnight at 4 °C. Beads were then cleaned using a washing buffer, and the immunoprecipitated RNA was subjected to RT-qPCR test for analysis. The experiments were conducted according to a previous study [23]. The primer sequences used for the ChIP assay are listed in Supplementary Table S5.

### Glutathione-S-transferase (GST)-pull-down assay

*Escherichia coli* was used to clone and express the GST-tagged YAP1 prokaryotic plasmids. GST-beads (Mabnus; cat. M7006) were used to obtain and purify GST-YAP1 fusion proteins, as described in our earlier work [11], and His-tagged CPNE3 was purified on a Ni–NTA column (Thermo Fisher Scientific; with 0.5 M imidazole as the elution solvent). The GST-YAP1 fusion protein or GST-control was introduced into an Eppendorf tube along with purified His-tagged CPNE3. Protein-bound GST-agarose beads were subjected to WB after three washes with GST lysis buffer.

### Ubiquitination assay

Plasmids expressing myc-β-TRCP, Flag-YAP1, His-Ub, HA-CPNE3, or siCPNE3-#2 were transfected into BGC-823 cells for 48 h. Cells were exposed to 10 μM of MG132 for 6 h prior to lysis. At 4 °C for 24 h, the cell lysate was treated with anti-Flag polyclonal antibodies. Immunoprecipitated proteins were collected by boiling the beads and were examined by ubiquitin immunoblotting to identify ubiquitinated YAP [11]

### Clinical significance analysis

By mining cancer-related Gene Expression Profiling Interactive Analysis (GEPIA) datasets, bioinformatics techniques were employed to predict differences in *CPNE3* expression between GC and normal gastric tissues. The relationship between *CPNE3* mRNA expression and OS, post-progressive survival (PPS), and first progression (FP) in patients with GC was examined using the Kaplan–Meier plotter database. RT-qPCR, WB, and IHC staining were used to confirm the expression of CPNE3 in GC and nearby normal tissues. The URLs used in this study are listed in Supplementary Table S6.

### Statistical analysis

A minimum of three separate iterations were performed for each experiment. The National Institutes of Health ImageJ program was used to measure the strength of the WB bands and fluorescence signals. GraphPad Prism 9 software (GraphPad, USA) was used to perform unpaired two-tailed Student's t-tests to evaluate the data. Statistical significance was considered when *p* < 0.05, using the SPSS 19.0 program (IBM, USA) for statistical analyses.

## Results

### Screening of *CPNE3* as a downstream target gene regulated by YAP1

**Fig. 1:**
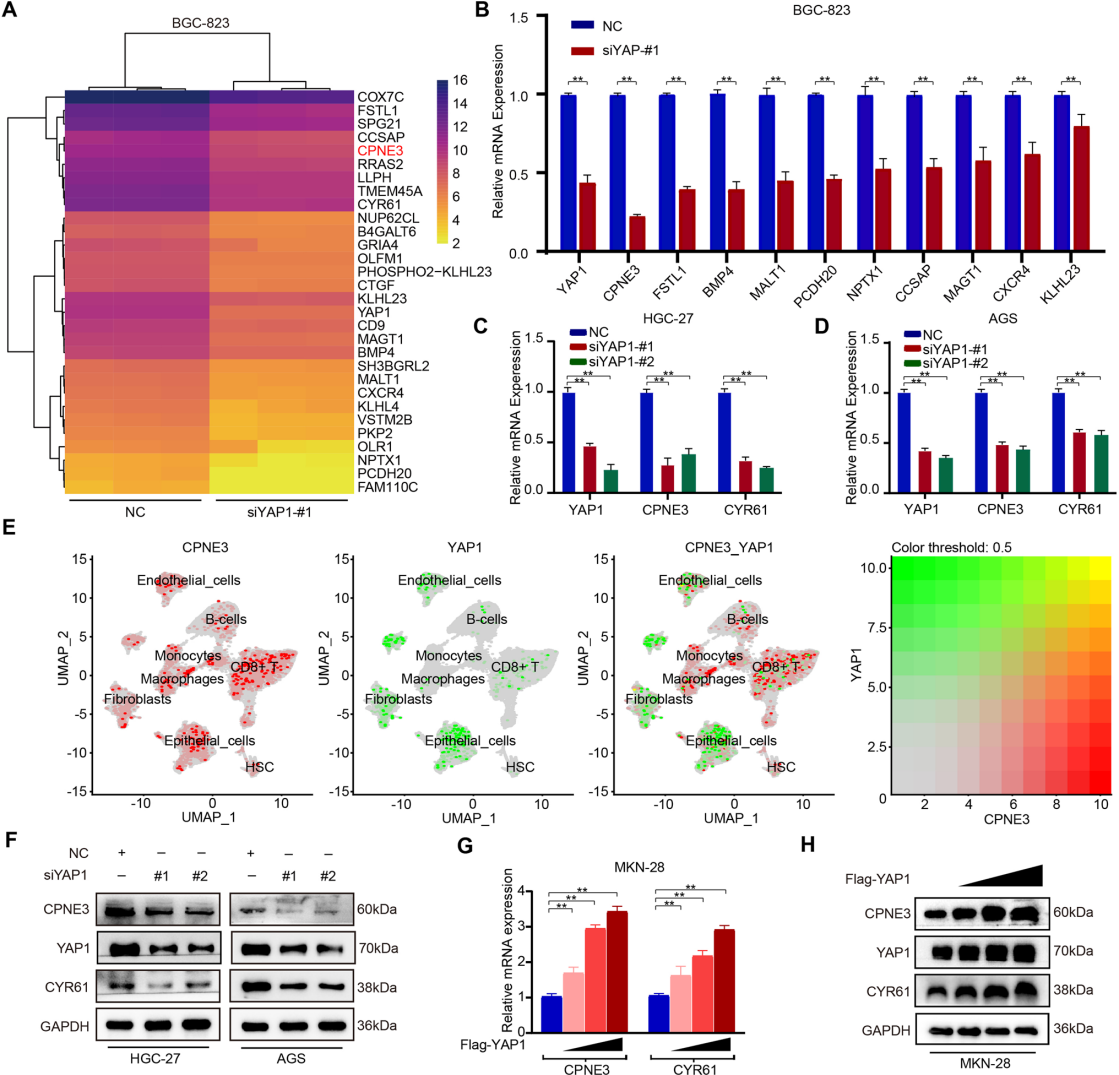
Screening of *CPNE3* as a downstream target gene regulated by YAP1. **A** mRNA sequencing in BGC-823 cells with downregulated YAP1 expression. **B** Examination of mRNA expression by quantitative reverse transcription polymerase chain reaction (RT-qPCR) after downregulating YAP1 in BGC-823 cells. **C**, **D** mRNA expression levels of *YAP1*, *CPNE3*, and *CYR61* in AGS and HGC-27 cells (transfected with NC, siYAP1-#1, and siYAP1-#2 siRNAs). **E** Two-dimensional visualization of CPNE3 and YAP1 in single-cell clusters in patients with gastric cancer (GC). **F** Protein expression levels of YAP1, CPNE3, and CYR61 following down-regulation of YAP1 expression in AGS and HGC-27 cells. **G**, **H** After gradient overexpression of Flag-YAP1, the YAP1, CPNE3, and CYR61 proteins and mRNA levels were detected by western blotting (WB) and RT-qPCR, respectively. Three independent biological experiments were performed, and statistical significance is denoted by * *p* < 0.05 and ** *p* < 0.01

To screen new downstream target genes of the YAP1 pathway, we downregulated the expression of YAP1 in the BGC-823 cell line by siRNA and then performed mRNA sequencing to identify the candidate genes (Fig. 1a), among these genes, 11 genes were screened out from the top 30 genes with a fold change difference > 3 (*p* < 0.05). Subsequently, RT-qPCR results indicated that the downregulation of YAP1 in BGC-823 cells significantly reduced the mRNA expression of certain genes, among which *CPNE3* was the most downregulated (Fig. 1b).

To explore the role of CPNE3 in GC, we analyzed the mRNA expression levels using the GEPIA database and found that *CPNE3* mRNA was highly expressed in multiple cancers (Supplementary Fig. S1A). Additionally, we used the Gene Expression Omnibus (GEO) database to extract primary GC data (GSE183904) for single-cell data analysis. The results showed that CPNE3 and YAP1 in the single-cell transcriptome Uniform Manifold Approximation and Projection (UMAP) was co-expressed in epithelial cells (Fig. 1e, Supplementary Fig. S1B).

We further verified that downregulation of YAP1 reduced the expression of *CPNE3* and *CYR61* at the mRNA and protein levels in HGC-27 and AGS cells (Fig. 1c, d, f, Supplementary Fig. S2A). In contrast, CPNE3 caused a dose-dependent increase in YAP1 overexpression (Fig. 1g, h, Supplementary Fig. S2B), suggesting that *CPNE3* may be a direct YAP1 target gene.

### YAP1/TEADs directly regulates CPNE3 expression

**Fig. 2:**
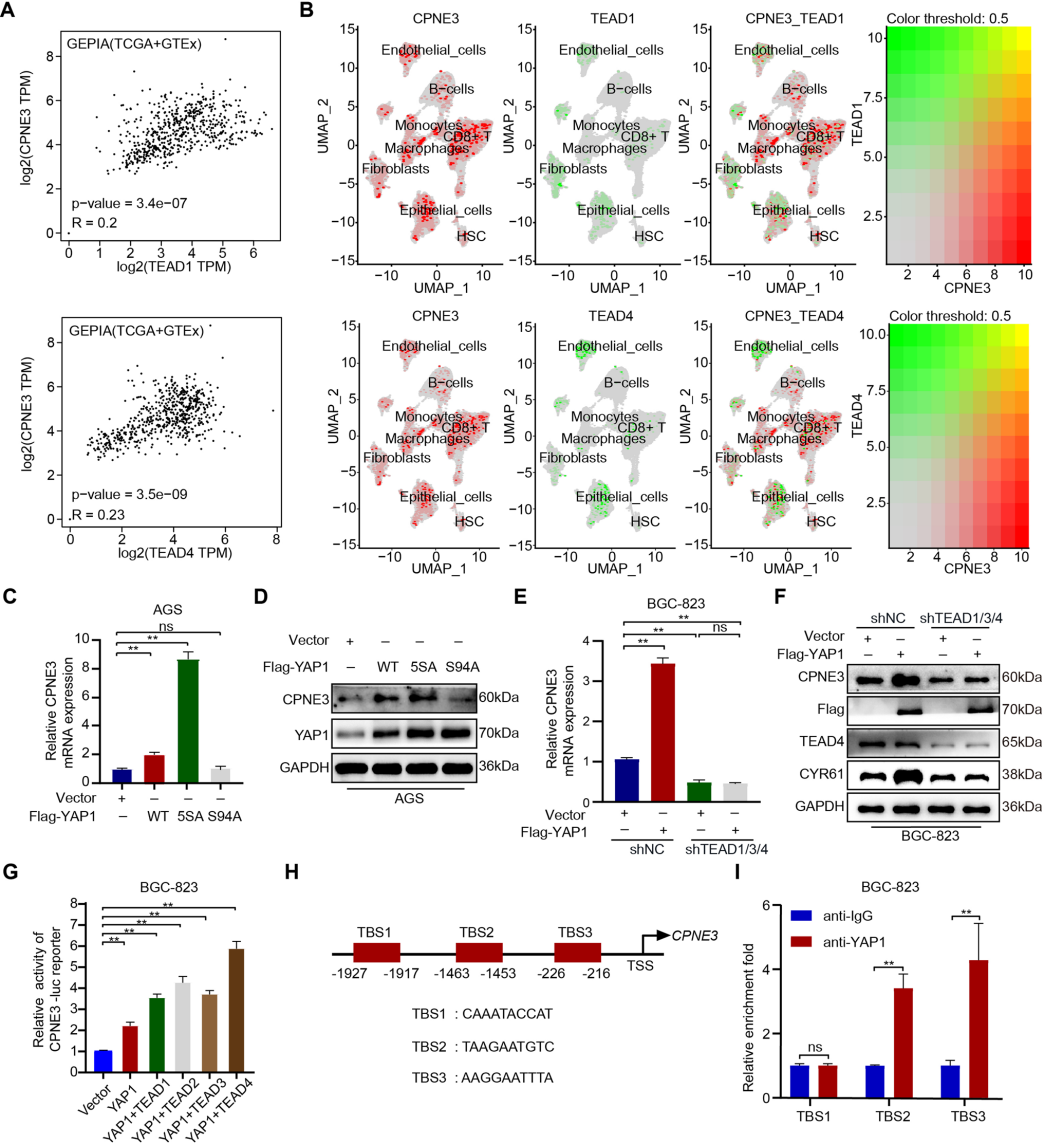
Direct regulation of CPNE3 expression by YAP1/TEAD4. **A** *CPNE3* mRNA levels were analyzed with TEAD1 and TEAD4 expression using the GEPIA database. **B** Two-dimensional visualization of CPNE3 and TEAD1 or TEAD4 in single-cell clusters in patients with GC. **C**, **D** *CPNE3* mRNA was detected by RT-qPCR, and expression of CPNE3 and YAP1 by WB with appropriate antibodies as indicated in AGS cells stably expressing YAP1-WT, YAP-5SA, or YAP-S94A. **E**, **F** YAP1-WT plasmid was introduced into TEADs-deficient BGC-823 cells, and *CPNE3* mRNA expression and protein expression levels were detected by RT-qPCR and WB. **G** The *CPNE3* promoter was cloned into a luciferase reporter to verify the effect of YAP1 and TEAD1/2/3/4 on *CPNE3* transcriptional activity. **H** The JASPAR database yielded several possible TEAD4 binding sites on the *CPNE3* promoter. **I** Three primers created for the aforementioned TEAD4-binding sites were used for chromatin immunoprecipitation

Since YAP1 mainly pairs with the transcription factor family of TEADs to exert transcriptional regulation, we first investigated the relationship between *CPNE3* and the TEADs family. Gene co-expression analysis performed using the GEPIA database revealed that *CPNE3* positively correlated with TEAD1 and TEAD4 expression at the mRNA level (Fig. 2a). Moreover, our results showed that CPNE3 and TEAD1, and TEAD4 were co-expressed in epithelial cells of the UMAP single-cell transcriptome (Fig. 2b, Supplementary Fig. S1C). The introduction of exogenous YAP1-wild type and YAP-5SA (an activating mutant) significantly increased CPNE3 expression in AGS cells, whereas YAP-S94A, a mutant lacking the ability to activate TEADs, had no remarkable influence on expression CPNE3 (Fig. 2c, d, Supplementary Fig. S2C). Then, we established stable TEAD-deficient BGC-823 cells with ShRNA treatment and introduced the YAP1-WT plasmid into the TEAD-deficient BGC-823 cells. RT-qPCR and WB results indicated that CPNE3 expression was no longer induced by YAP1 overexpression (Fig. 2e, f, Supplementary Fig. S2D). These results indicate that YAP1 induces the expression of CPNE3 and that this function may be dependent on TEADs.

Subsequentially, we cloned the *CPNE3* promoter into a basic luciferase reporter to verify the effects of YAP1 and TEAD1/2/3/4 on *CPNE3* transcriptional activity. As shown in Fig. 2g, dual-luciferase assays suggested that overexpression of YAP1 and TEAD1/2/3/4 significantly enhanced luciferase activity driven by the *CPNE3* promoter. We searched the *CPNE3* promoter region on the National Center for Biotechnology Information website and predicted multiple potential TEAD4-binding sites (TBS) on the *CPNE3* promoter using the JASPAR database. The three sites with the highest scores are shown in Fig. 2h. To further confirm these predictions, we performed ChIP analysis with three primers designed for the TBS and found that Flag-YAP1 showed extensive binding to the TBS2 and TBS3 regions (Fig. 2i). In summary, these results suggest that *CPNE3* is a target gene directly regulated by the YAP1/TEADs complex.

### CPNE3 promotes proliferation and chemotherapy resistance in GC cells

**Fig. 3:**
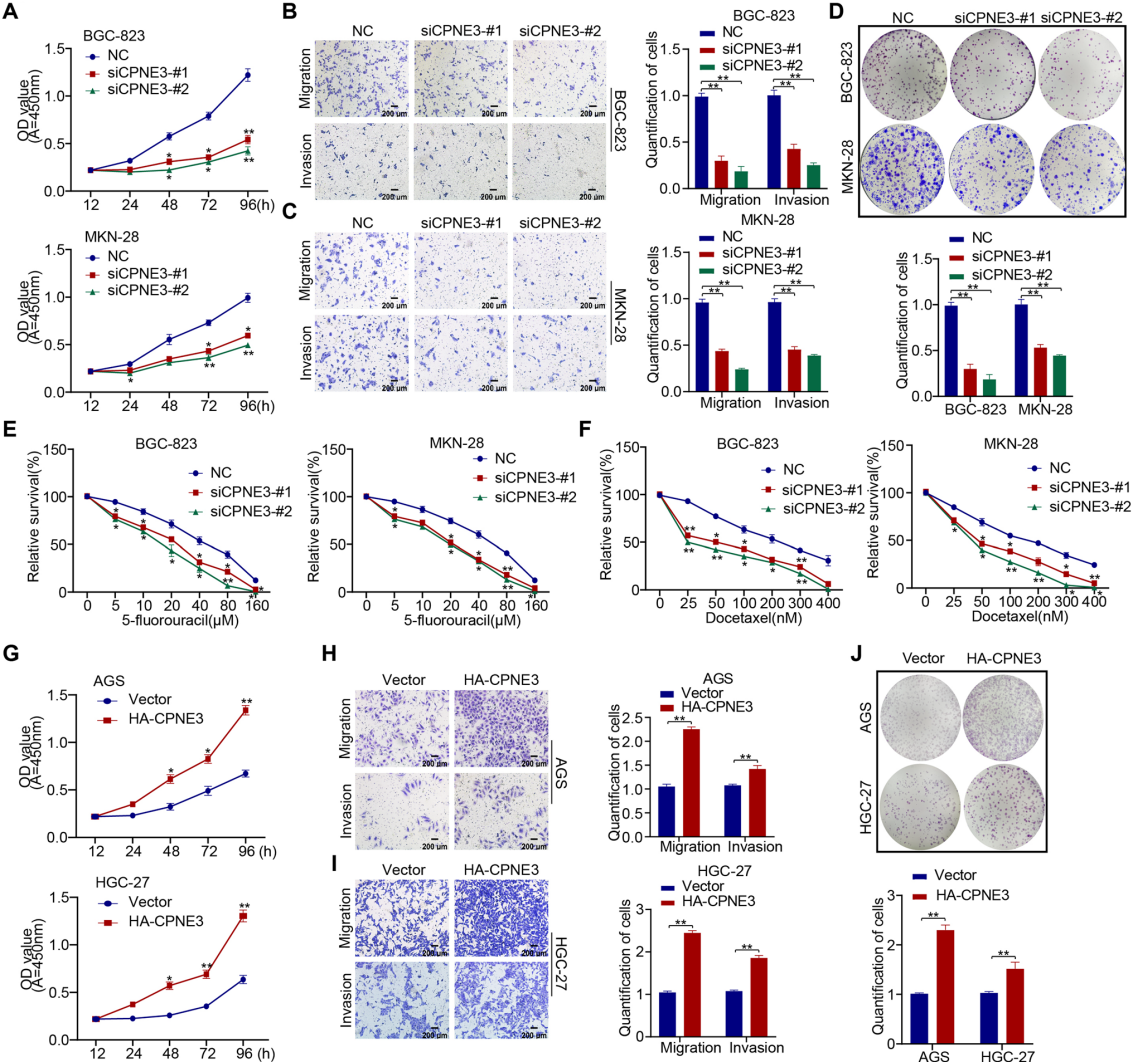
Mechanism of CPNE3 in promoting proliferation and drug resistance in vitro in GC cells. **A** The proliferation of BGC-823 and MKN-28 cells was significantly inhibited when CPNE3 was downgraded using siCPNE3-#1 or siCPNE3-#2, as shown by the CCK-8 assay. **B**–**D** Transwell assay and clone formation assay revealed suppressed cell migration, invasion, and colony formation ability of GC cells. **E**, **F** In vitro chemosensitivity tests using gradient concentrations of 5-fluorouracil or docetaxel showed that downregulation of CPNE3 enhanced the sensitivity of GC cells to chemoresistant drugs. **G**–**J** HA-CPNE3 plasmids were stably transfected into AGS and HGC-27 cells, and cell function tests demonstrated that HA-CPNE3 significantly increased GC cell proliferation, migration, invasion, and colony formation ability. Three independent biological experiments were conducted, which consistently yielded similar results. Statistical significance is indicated by *p < 0.05 and **p < 0.01. Scale bar: 200 μm

To further clarify the biological function of CPNE3 in GC, we first downregulated CPNE3 expression in BGC-823 and MKN-28 cell lines based on the CPNE3 protein levels in various GC cell lines. According to the CCK-8 assay, the downregulation of CPNE3 remarkably inhibited the proliferation of GC cells (Fig. 3a). Transwell assays revealed that the ability of cells with downregulated CPNE3 to migrate and invade was much weaker than that of the control group (Fig. 3b, c). Colony formation experiments indicated that the downregulation of CPNE3 dramatically reduced cell proliferation (Fig. 3d). We also investigated how the downregulation of CPNE3 affects the sensitivity of GC cells to chemotherapy. As shown in Fig. 3e, f, downregulation CPNE3 greatly increased the sensitivity of BCC-823 and MKN-28 cells to docetaxel and 5-fluorouracil.

Then, we investigated the biological functions of HGC-27 and AGS cells with upregulated CPNE3 expression levels. HGC-27 and AGS cells transfected with the HA-CPNE3 plasmid demonstrated greater cell proliferation, clone formation, migration, and invasion capabilities than the control group (Fig. 3g–j). To investigate whether CPNE3 plays a role in normal gastric epithelial cells, we downregulated CPNE3 expression in GES-1 cells using siRNA and performed cellular assays (Supplementary Figs. S2J, S3A). The results indicated that downregulation of CPNE3 slightly affected the proliferation, migration, invasion, and chemosensitivity of GES-1 cells (Supplementary Fig. S3B–E).

### CPNE3 regulates the Hippo-YAP1 signaling pathway

**Fig. 4:**
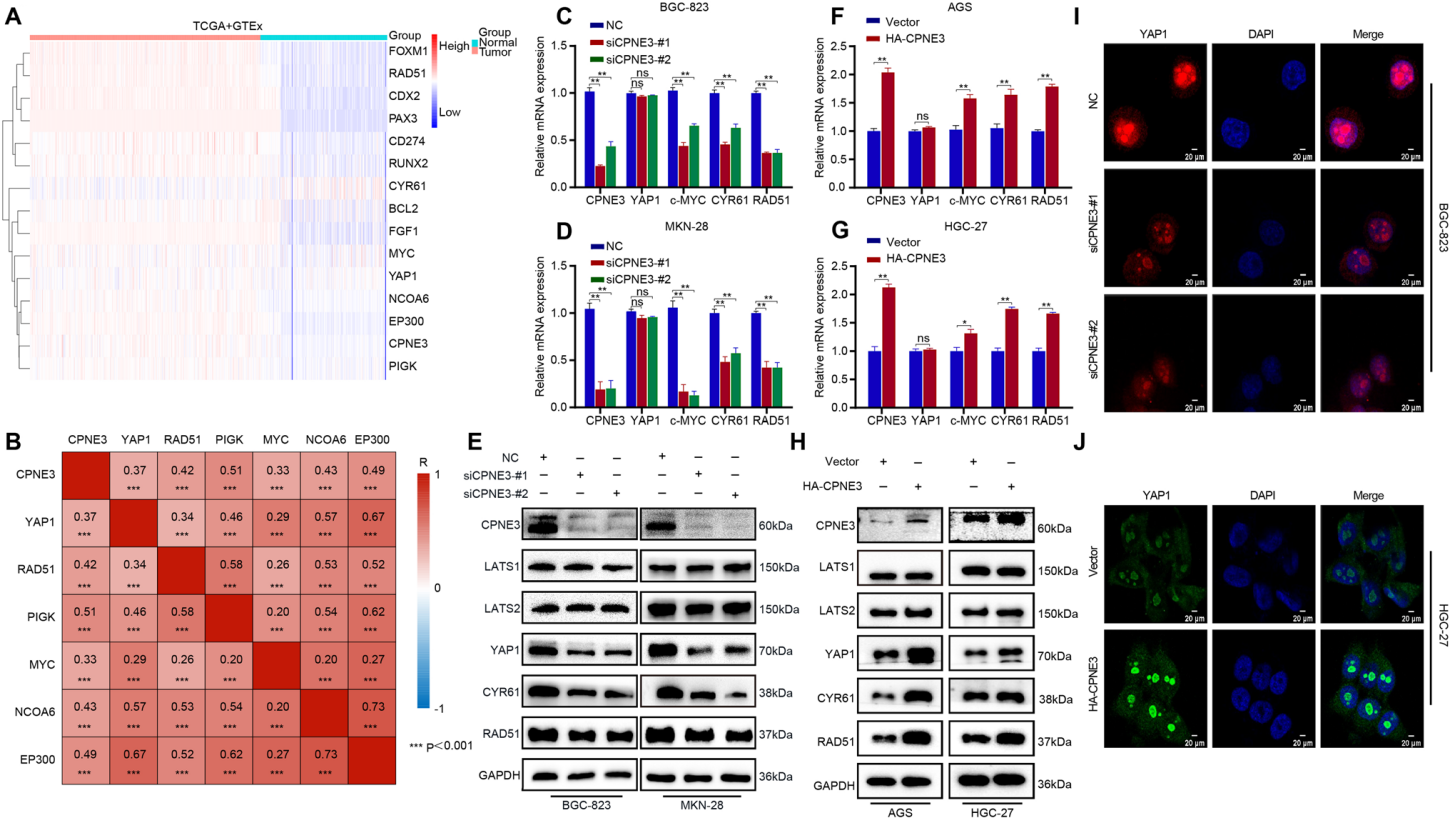
CPNE3 is associated with the Hippo-YAP1 pathway in GC. **A** Enrichment analysis of *CPNE3* with Hippo-YAP1. **B** Pearson correlation analyses of *CPNE3* with Hippo-YAP1 **C**–**E** Using siRNA, the expression of *CPNE3* at the mRNA and protein levels were downregulated in BGC-823 and MKN-28 cells. **F**, **G** Up-regulation of *CPNE3* expression by transfection of HA-CPNE3 plasmid in AGS and HGC-27 cells. **H** WB revealed that the expression of YAP1 and its target gene CYR61 were both considerably upregulated in AGS and HGC-27 cells using the same technique used to boost CPNE3 expression as above. **I**, **J** Confocal images of HA-CPNE3 plasmid transfection in HGC-27 cells and siCPNE3-#1 or siCPNE3-#2 transfection in BGC-823 cells with YAP1 labeling

To further understand the molecular mechanism by which CPNE3 regulates the malignant development of GC, we explored the correlation between CPNE3 and the Hippo-YAP1 pathway. Accordingly, we gathered the data of patients with GC from the Cancer Genome Atlas and the Genotype-Tissue Expression databases for differential expression and Pearson correlation analyses. The results of these analyses revealed that the expression of *CPNE3*, *YAP1*, *RAD51*, *PIGK*, *MYC*, *NCOA6*, and *EP300* was higher in tumor tissues than in normal tissues, and *CPNE3* expression was positively correlated with *YAP1*, *RAD51*, *PIGK*, *MYC*, *NCOA6*, and *EP300* expression in patients with GC (Fig. 4a, b), suggesting that CPNE3 may positively regulate the Hippo-YAP1 signaling pathway.

To more strongly support CPNE3' s regulatory function within the Hippo-YAP1 signaling cascade, we used siRNA in BGC-823 and MKN-28 cells to inhibit the expression of *CPNE3* mRNA. CYR61 and RAD51 have been recognized as YAP1 downstream target genes [24, 25], so we chose to use CYR61 and RAD51 as positive controls for regulating the YAP pathway. RT-qPCR analysis showed that downregulation of *CPNE3* expression in GC cells decreased *CYR61* and *RAD51* mRNA expression (Fig. 4c, d), and the opposite result was observed for *CPNE3* overexpression in HGC-27 and AGS cells (Fig. 4f, g). Furthermore, WB indicated that the down-regulation of CPNE3 expression resulted in a remarkable reduction in the protein levels of YAP1 and its target genes CYR61 and RAD51, whereas the up-regulation of CPNE3 showed the opposite effect. Notably, the expression of the YAP1 upstream regulators LATS1/2 demonstrated no discernible effect (Fig. 4e, h, Supplementary Fig. S2E, F). Additionally, immunofluorescence experiments revealed that CPNE3 promoted YAP1 enrichment in the nucleus (Fig. 4i, j). These findings indicate that the Hippo-YAP1 signaling pathway is regulated by CPNE3.

### CPNE3 stabilizes YAP1 by competitively binding YAP1 with β-TRCP

**Fig. 5:**
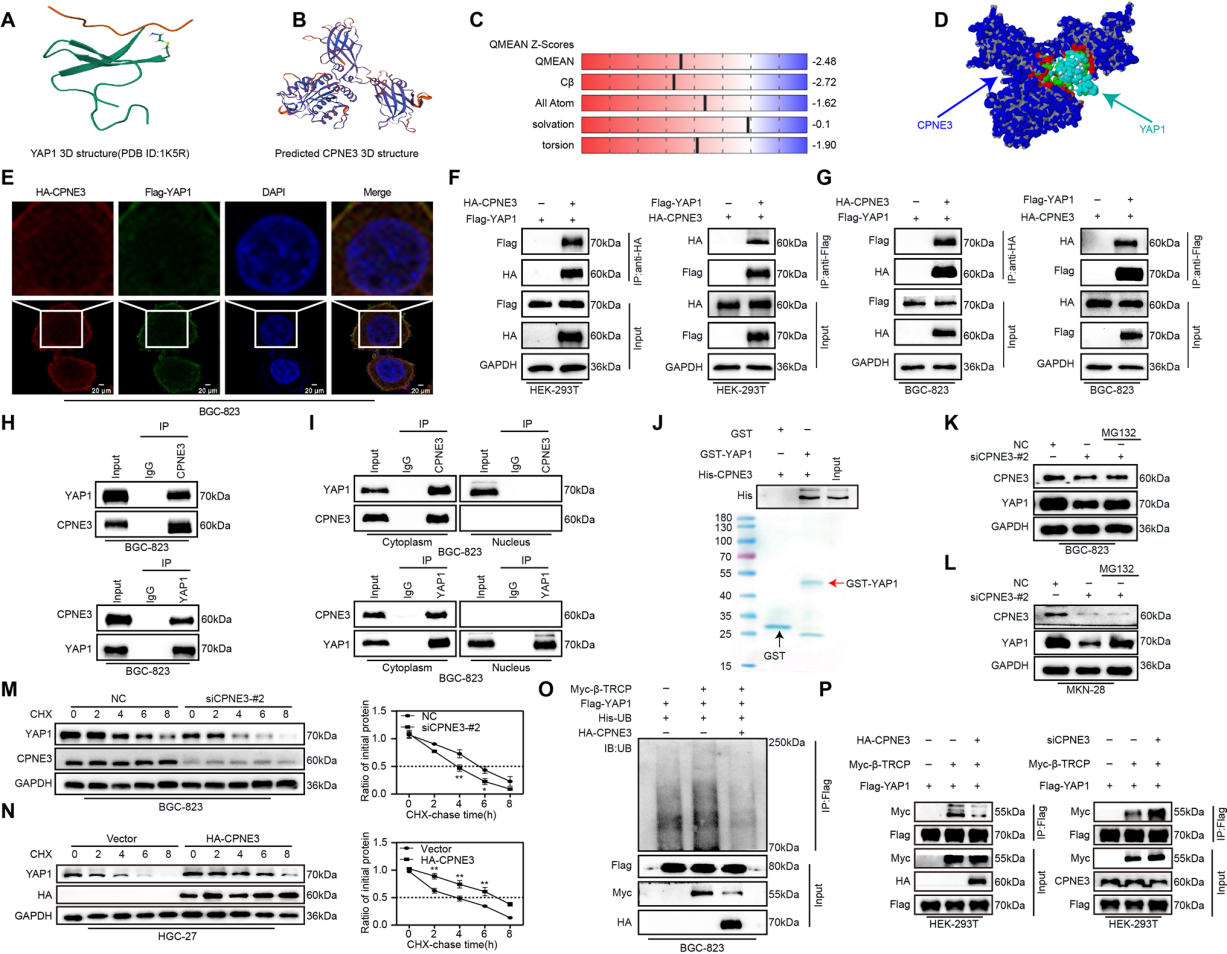
CPNE3 stabilizes YAP1 by competing with β-TRCP for binding to YAP1. **A** The three-dimensional structure of YAP1 from the Protein Data Bank database. **B**, **C** A predictive three-dimensional structural model and model quality evaluation of CPNE3 that was acquired from the Swiss-model database. **D** The anticipated interface of one of the complexes created when CPNE3 and YAP1 combine. **E** Immunofluorescence staining for HA-CPNE3 and Flag-YAP1 in BGC823 cells. **F**, **G** Results of CPNE3 or YAP1 co-immunoprecipitation (Co-IP) tests carried out in HEK-293 T and BGC-823 cells. **H** In BGC-823 cells, endogenous Co-IP was performed to evaluate the interaction between CPNE3 and YAP1. **I** In the cytoplasmic and nuclear protein lysates of BGC-823 cells, the interaction between endogenous CPNE3 and YAP1 was examined independently. **J** By using glutathione-S-transferase (GST)-pull-down assay, it was shown that His-CPNE3 and GST-YAP1 fusion proteins directly bind to one another. **K**, **L** Downregulation of CPNE3 in BGC-823 and MKN-28 cells decreases the expression of YAP1, while proteasome inhibitors MG132 reversed this process. **M**, **N** By using the cycloheximide test in BGC-823 cells transfected with siCPNE3-#2 and HGC-27 cells transfected with HA-CPNE3 plasmids, the impact of CPNE3 expression on the stability of YAP1 was evaluated. **O** Impact of HA-CPNE3 on the ubiquitination of YAP1 was assessed using a BGC-823 cell-based ubiquitination assay. **P** HEK-293 T cells were transfected with HA-CPNE3, Flag-YAP1, and myc-β-TRCP plasmids, and Co-IP was used to determine the binding status among the three

We explored possible binding between CPNE3 and YAP1 using several proteomic databases. First, we collected the 3D structure of YAP1 from the PDB database and predicted the 3D structure of CPNE3 from the Swiss-Model Repository database to confirm the link between CPNE3 and YAP1 (Fig. 5a, b). The prediction model was of excellent quality in terms of the assessment of quality, template matching, protein size, and features of amino acid residues, and could accurately depict the spatial structure of the CPNE3 protein (Fig. 5c, Supplementary Fig. S1D). Subsequent molecular docking using ZDOCK for the 3D structures of CPNE3 and YAP1 using ZDOCK revealed that CPNE3 and YAP1 formed a stable protein complex (Fig. 5d).

To further verify the interaction between CPNE3 and YAP1, we performed immunofluorescence experiments to examine the distribution of exogenous YAP1 and CPNE3 in BGC-823 cells, which showed co-localization with CPNE3 and YAP1 in the cytoplasm (Fig. 5e). Next, exogenous Flag-YAP1 and HA-CPNE3 plasmids were co-transfected into HEK-293 T and BGC-823 cells and the binding of CPNE3 to YAP1 was confirmed by Co-IP analysis (Fig. 5f, g). The same conclusions were drawn from the endogenous Co-IP assay (Fig. 5h), and cytoplasmic separation experiments showed that CPNE3 bonded to YAP1 in the cytoplasm but not in the nucleus (Fig. 5i). Moreover, we purified GST-YAP1 and His-CPNE3 proteins and demonstrated their direct binding using GST-pull down assays (Fig. 5j).

YAP1 undergoes various posttranslational modifications, and ubiquitination-proteasome degradation plays a key role in regulating YAP1 expression [26]. To verify the role of CPNE3 in maintaining YAP1 stability, we used an siRNA to downregulate CPNE3 expression in BGC-823 and MKN-28 cells. WB indicated that CPNE3 depletion reduced YAP1 protein expression, whereas the proteasome inhibitor MG132 reversed this process (Fig. 5k, l). Half-life analysis revealed that YAP1 was more unstable in CPNE3-deficient cells (Fig. 5m). In contrast, CPNE3 overexpression significantly prolonged the half-life of YAP1 in the HGC-27 cells (Fig. 5n). Furthermore, ubiquitination experiments revealed that overexpression of CPNE3 reduced the ubiquitination level of YAP1 (Fig. 5o), suggesting that CPNE3 is involved in inhibiting YAP1 ubiquitination to maintain YAP1 stability.

Previous research has shown that β-TRCP is a ubiquitin E3 ligase that plays an important role in YAP1 ubiquitination by binding to YAP1 protein [27]. Therefore, we hypothesized that CPNE3 competes with β-TRCP for binding to YAP1, thereby inhibiting the subsequent ubiquitination of YAP1 protein. We further examined the effect of CPNE3 on the combination of β-TRCP and YAP1, and found that overexpression of CPNE3 inhibited the combination of β-TRCP to YAP1 in HEK-239 T cells. In contrast, down-regulation of CPNE3 expression increased the binding of β-TRCP to YAP1 (Fig. 5p). Together, these results demonstrate that CPNE3 reduces the ubiquitination and degradation of YAP1 by direct binding to YAP1.

### CPNE3 promotes GC progression in a partial YAP1-dependent manner

**Fig. 6:**
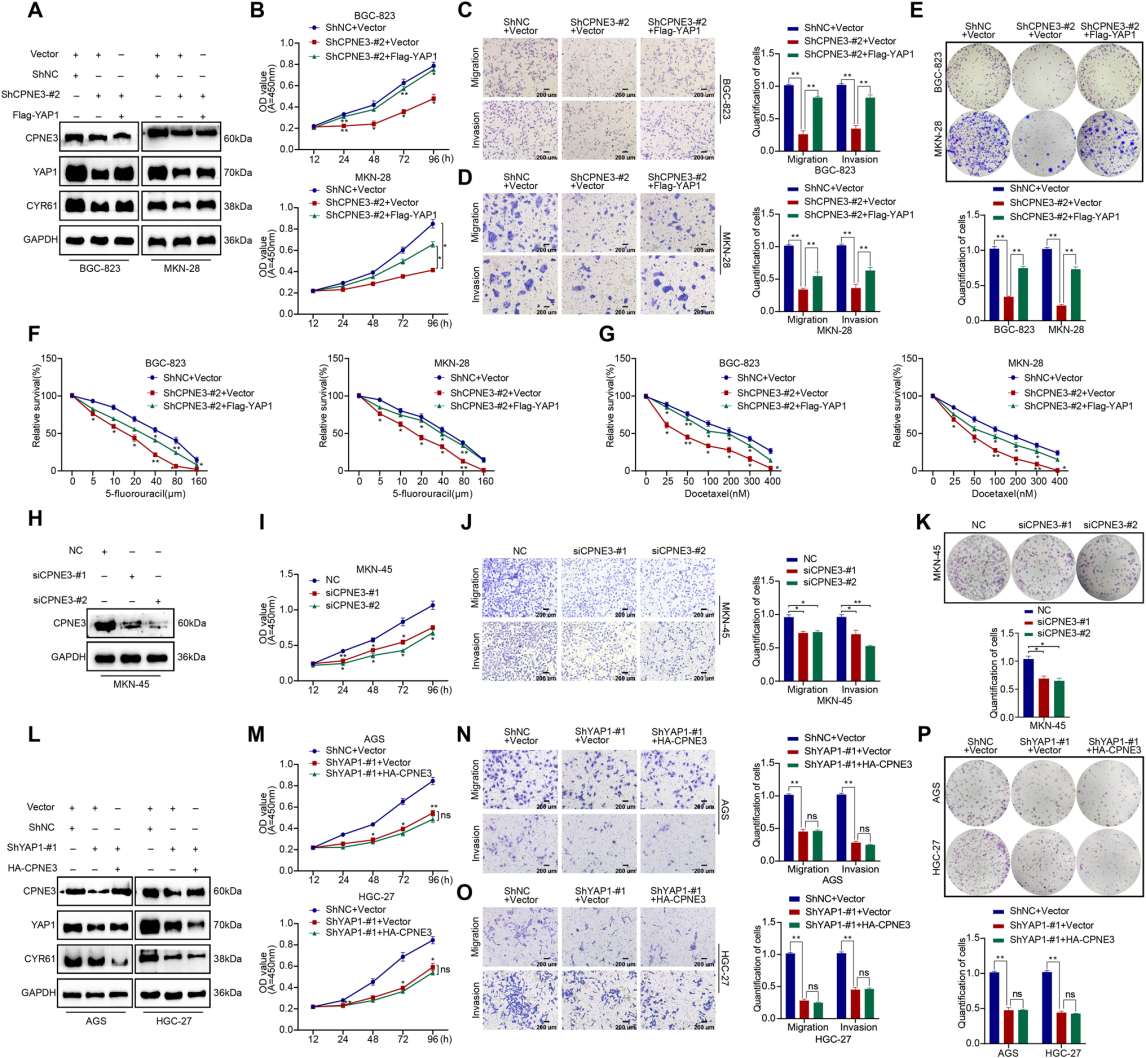
CPNE3 promotes GC progression in a partial YAP1-dependent manner. BGC-823 and MKN-28 cells were treated with the ShCPNE3-#2 plasmid to downregulate the expression of CPNE3 and the Flag-YAP1 plasmid to concurrently increase the expression of YAP1. **A** WB was used to measure the expression of CPNE3, YAP1, and CYR61. **B**–**G** Phenotyping assays were used to determine the degree to which the CPNE3 depletion-induced suppression of GC cell proliferation, migration, invasion, colony formation, and drug resistance could be reversed by the overexpression of exogenous YAP1. **H**–**K** The capacity of MKN-45 cells to proliferate, invade, migrate, and form colonies was weakly inhibited by downregulation of CPNE3 expression in MKN-45 cells lacking YAP1 expression. ShYAP1-#1 plasmid-mediated stable *YAP1* knockdown or HA-CPNE3 plasmid-mediated simultaneous overexpression of CPNE3 in AGS and HGC-27 cells. **L** WB was used to investigate the relevant protein levels. **M**–**P** The overexpression of CPNE3 did not alleviate the suppression of GC cell proliferation, migration, invasion, and colony formation brought on by the downregulation of YAP1. Three independent biological experiments were conducted, and statistical significance is shown by the notations, *p < 0.05 and **p < 0.01

Our previous study revealed that CPNE3 promoted the malignant behavior of GC cells and maintained YAP1 stability. Therefore, it was necessary to determine whether the biological characteristics of CPNE3 are dependent on YAP1 expression. We carried out a function-reversal experiment, and WB showed that the overexpression of Flag-YAP1 in BGC-823 and MKN-28 cells reversed the decrease in CYR61 caused by CPNE3 deletion (Fig. 6a, Supplementary Fig. S2G). CCK-8 assay showed that exogenous YAP1 reversed the proliferative capability of CPNE3-silenced BGC-823 and MKN-28 cells (Fig. 6b). Moreover, the effects of CPNE3 deletion on the invasion, migration, and colony-forming capabilities of BGC-823 and MKN-28 cells were partly reversed by the overexpression of exogenous YAP1 (Fig. 6c–e), suggesting that the biological function of CPNE3 is at least partially dependent on YAP1. In addition, increased resistance of CPNE3-silenced BGC-823 and MKN-28 cells to 5-fluorouracil or docetaxel was observed when YAP1 was upregulated (Fig. 6f, g). To further verify these results, we repeated the experiment in YAP1-deficient MKN-45 cells (Fig. 6h, Supplementary Fig. S2H), and found that the downregulation of CPNE3 only slightly inhibited the proliferation, invasion, migration, and colony-forming abilities of YAP1-deficient MKN-45 cells (Fig. 6i–k).

To confirm our findings, we performed blockade experiments, which showed that transfection of the HA-CPNE3 plasmid into *YAP1*-silenced HGC-27 and AGS cells failed to improve CYR61 levels (Fig. 6l, Supplementary Fig. S2I). The CCK-8 results showed that when YAP1 was silenced, the ability of CPNE3 to promote the proliferation of HGC-27 and AGS cells was significantly inhibited (Fig. 6m). Additionally, the reduced invasion, migration, and colony-forming abilities of AGS and HGC-27 cells were not enhanced by CPNE3 overexpression (Fig. 6n–p). These results suggest that CPNE3 plays a key role in promoting the malignant progression of GC in a partially YAP1-dependent manner.

### CPNE3 promotes the growth of GC xenograft tumors in vivo

**Fig. 7:**
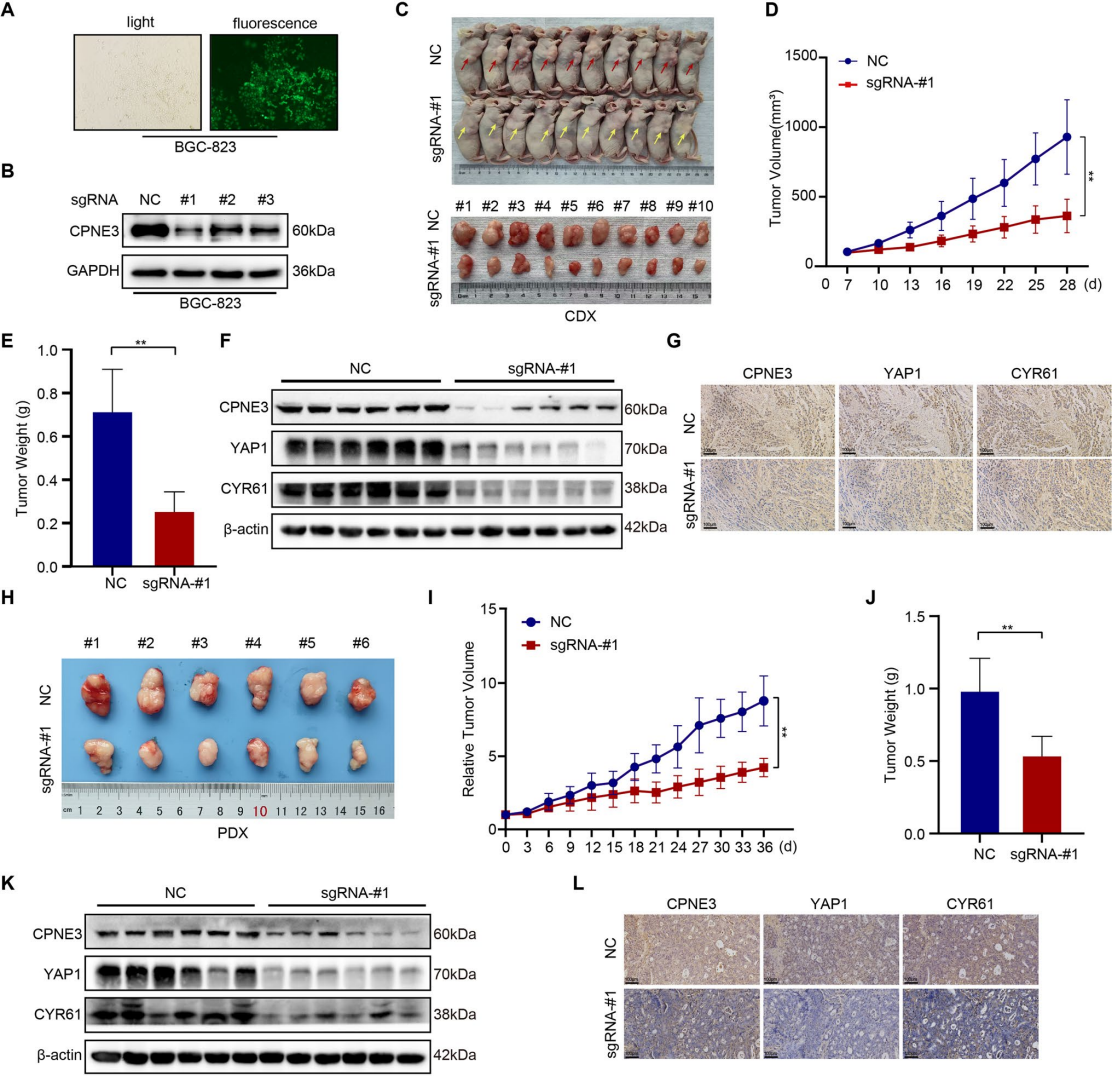
CPNE3 promotes GC growth in vivo. **A**, **B** Cell line with stable silencing of CPNE3 expression was established in BGC-823 cells by infection with lentivirus encoding sgRNA targeting CPNE3 or negative control. **C**–**E** Silencing of CPNE3 significantly reduced tumor growth in vivo, and the weight and volume of the tumor tissues were significantly lower than those in controls (mean ± standard error of the mean [SEM] n = 10/group). **F**, **G** Immunohistochemistry (IHC) and WB tests were used to assess the protein levels of CPNE3, YAP1, and CYR61 in tumor tissues from subcutaneous cell line-derived xenograft models constructed from BGC-823 cells with CPNE3 expression downregulated by lentivirus. **H**–**J** Using the same method described above to validate the function of CPNE3 in a patient-derived xenograft (PDX) model of GC, silencing CPNE3 significantly reduced tumor growth, weight, and volume in vivo (mean ± SEM n = 6/group). **K**, **L** Protein expression of CPNE3, YAP1, and CYR61 were detected using WB and IHC, assays in BGC-823 cells after stably downregulating CPNE3 expression in the PDX model

To further study the biological function of CPNE3 in vivo, we established CDX tumor models using Clustered Regularly Interspaced Short Palindromic Repeats (CRISPR)-associated protein 9 (CRISPR/Cas9). By infecting BGC-823 cells with a lentivirus encoding an sgRNA targeting CPNE3 (sgRNA-CPNE3) or a negative control (sgRNA-NC), CPNE3 was silenced. After verifying the effectiveness of the deletion using WB (Fig. 7a, b), the cells were injected subcutaneously into BALB/c nude mice. As shown in Fig. 7c, CPNE3 knockdown dramatically slowed tumor development in vivo (Fig. 7d, e), and the tumor weight of CDX (n = 10/group) models was significantly suppressed (mean ± standard error of the mean: sgRNA-CPNE3 vs. sgRNA-NC: 0.257 ± 0.028 vs. 0.716 ± 0.063; *p* < 0.01). Next, we used tumor tissues from the CDX model for IHC and WB. As shown in Fig. 7f, g, the expression levels of CPNE3, YAP1, and CYR61 were remarkably reduced in the tumor tissues of the CPNE3-silenced group.

To assess the therapeutic efficacy of targeting CPNE3 in a more predictive preclinical model, we validated the role of CPNE3 in a PDX model of GC. Intertumoral injection of lentivirus in BALB/c nude mice greatly slowed tumor development in the PDX model, which was consistent with the findings of earlier in vivo and in vitro experiments (Fig. 7h). The tumor growth rate of the PDX (n = 6/group) models significantly decreased after the intratumoral injection of the lentivirus encoding sgRNA-CPNE3, with the relative tumor proliferation rates value of 0.480 (mean relative tumor volume: sgRNA-CPNE3 vs. sgRNA-NC: 4.221 vs. 8.777; *p* < 0.01). Additionally, the weight and volume of the PDX model decreased after intratumoral injection of sgRNA-CPNE3 (Fig. 7i, j). Tumor tissues from the PDX models were analyzed by WB and IHC, and the results indicated that YAP1 and CYR61 protein levels significantly decreased when CPNE3 was downregulated (Fig. 7k, l). Collectively, these findings imply that high CPNE3 expression facilitates GC tumor growth in vivo.

### CPNE3 is an independent prognostic factor for poor prognosis in patients with GC

To explore the prognostic significance of CPNE3, we used the UALCAN and Kaplan–Meier plotter databases to analyze *CPNE3* mRNA expression, which indicated that *CPNE3* mRNA expression was considerably higher in GC tissues and was associated with poor OS, PPS, FP, and clinical stage in patients with GC (Supplementary Fig. S4A–E). Therefore, CPNE3 is a potential prognostic target in GC.

**Fig. 8:**
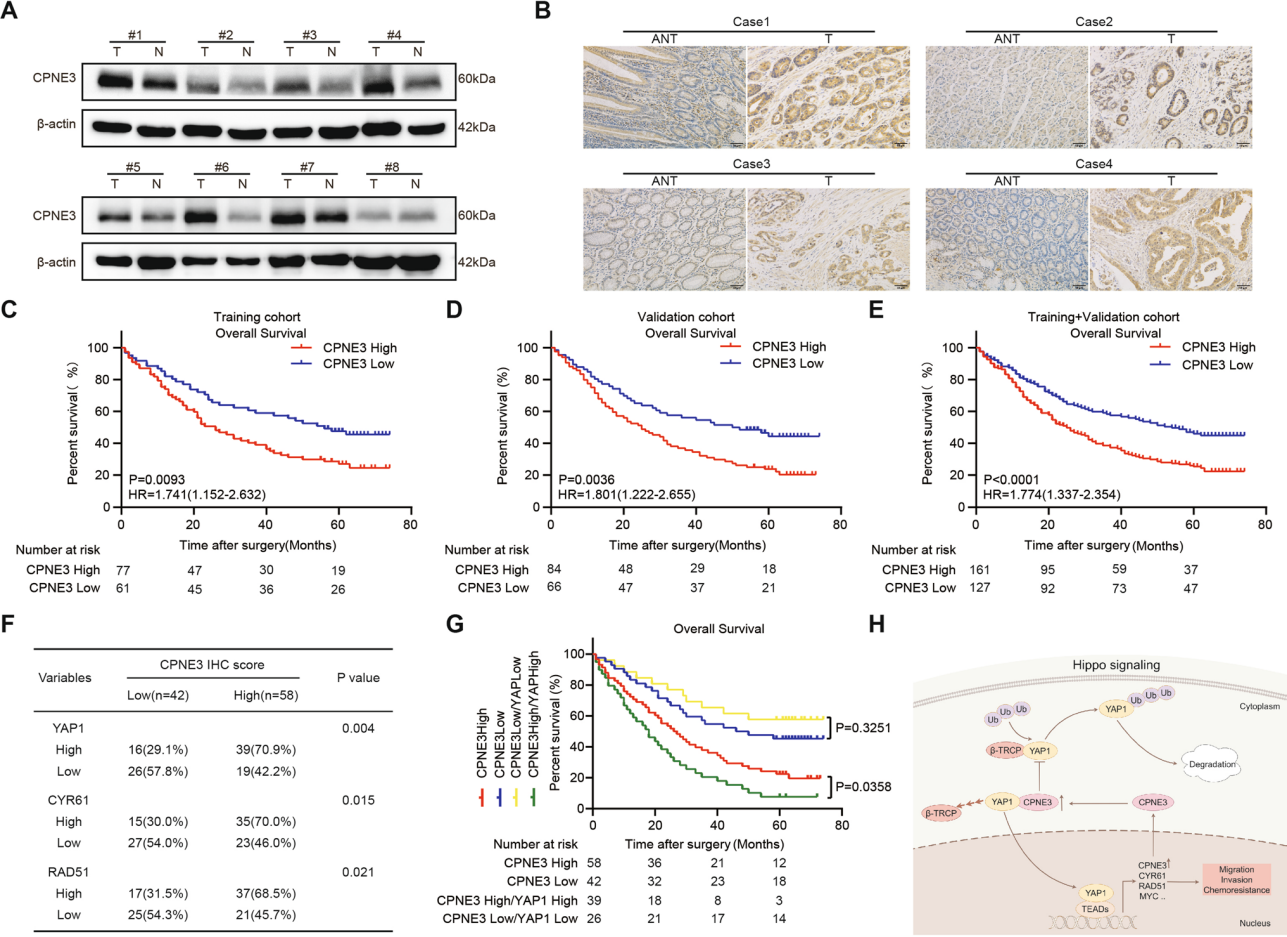
CPNE3 is an independent prognostic factor that causes poor prognosis in patients with GC. **A** Using Western blotting, the protein levels of CPNE3 in eight pairs of GC patient tissue samples were measured. **B** CPNE3 protein expression was examined the IHC of tumor tissues (n = 20) and matched normal tissues (n = 20) from patients with GC. **C**–**E** Overall survival and CPNE3 expression were used to stratify the patients in the training, validation, and training + validation groups, followed by Kaplan–Meier analysis. **F** The chi-square test was used to determine the relationship between the expression of CPNE3 and that of YAP1, CYR61, and RAD51. **G** Kaplan–Meier survival analysis was performed in 100 patients who were categorized based on their CPNE3 and YAP1 protein levels. **H** Diagram by Figdraw showing the process through which the CPNE3-YAP1 positive feedback loop promotes GC

Next, to verify the clinical significance of CPNE3 expression in GC tissues, we used WB to examine CPNE3 protein expression in eight pairs of fresh gastric tissues. The results showed that CPNE3 protein levels in GC tissues were significantly higher than those in the surrounding tissues (Fig. 8a). We performed IHC to analyze CPNE3 protein expression in tumor tissues (n = 20) and paired normal tissues (n = 20) from patients with GC. The findings demonstrated that CPNE3 protein expression was significantly higher in GC tissues than in paired normal tissues (Fig. 8b).

A total of 288 patients with GC were divided into training (n = 138) and validation (n = 150) cohorts, and their prognostic data were evaluated. Analysis of clinicopathological features is presented in Table 1. In the training cohort, patients with high CPNE3 expression had a substantially shorter median survival time (median survival time [MST] = 26 months) than those with low CPNE3 expression (MST = 56 months; *p* = 0.0093) (Fig. 8c). The depth of local infiltration, lymph node metastasis, TNM stage, and high CPNE3 expression were prognostic variables in GC according to the findings of univariate Cox regression analysis (Table 2). Furthermore, multivariate regression research revealed that, CPNE3 (hazard ratio [HR] = 1.555,95% confidence interval [CI]: 1.003–2.412, *p* = 0.049), and TNM stage (HR = 0.211, 95% CI 0.095–0.467, *p* < 0.001) were an independent prognostic factor (Table 2).

**Table 1:** Clinical characteristics of patients according to CPNE3 in training, validation and training + validation cohort

Variables	Training cohort (n = 138)				Validation cohort (n = 150)				Training + Validation cohort (n = 288)			
	N	Low CPNE3 (%)	High CPNE3 (%)	*p* value	N	Low CPNE3 (%)	High CPNE3 (%)	*p* value	N	Low CPNE3 (%)	High CPNE3 (%)	*p* value
Gender				0.935				0.591				0.656
Male	91	40 (44.0%)	51 (56.0%)		90	38 (42.2%)	52 (57.8%)		181	78 (43.1%)	103 (56.9%)	
Female	47	21 (44.7%)	26 (55.3%)		60	28 (46.7%)	32 (53.3%)		107	49 (45.8%)	58 (54.2%)	
Age (years)				0.663				0.937				0.807
< 60	65	30 (46.2%)	35 (53.8%)		71	31 (43.7%)	40 (56.3%)		136	61 (44.9%)	75 (55.1%)	
≥ 60	73	31 (42.5%)	42 (57.5%)		79	35 (44.3%)	44 (55.7%)		152	66 (43.4%)	86 (56.6%)	
Tumor size (cm)				0.171				0.750				0.473
≥ 4	61	23 (37.7%)	38 (62.3%)		66	30 (45.5%)	36 (54.5%)		127	53 (41.7%)	74 (58.3%)	
< 4	77	38 (49.4%)	39 (50.6%)		84	36 (42.9%)	48 (57.1%)		161	74 (46.0%)	87 (54.0%)	
Differentiation status				0.192				0.184				0.063
Poor and undifferentiated	94	38 (40.4%)	56 (59.6%)		93	37 (39.8%)	56 (60.2%)		187	75 (40.1%)	112 (59.9%)	
Well + Moderate	44	23 (52.3%)	21 (47.7%)		57	29 (50.9%)	28 (49.1%)		101	52 (51.5%)	49 (48.5%)	
Lauren type				0.266				0.989				0.437
Intestinal type	57	22 (38.6%)	35 (61.4%)		66	29 (43.9%)	37 (56.1%)		123	51 (41.5%)	72 (58.5%)	
Diffuse type	81	39 (48.1%)	42 (51.9%)		84	37 (44.0%)	47 (56.0%)		165	76 (46.1%)	89 (53.9%)	
Depth of invasion				0.033				0.036				0.003
T3 + T4	86	32 (37.2%)	54 (62.8%)		87	32 (36.8%)	55 (63.2%)		173	64 (37.0%)	109 (63.0%)	
T1 + T2	52	29 (55.8%)	23 (44.2%)		63	34 (54.0%)	29 (46.0%)		115	63 (54.8%)	52 (45.2%)	
Lymph node metastasis				0.048				0.047				0.005
N1 + N2 + N3	104	41 (39.4%)	63 (60.6%)		114	45 (39.5%)	69 (60.5%)		218	86 (39.4%)	132 (60.6%)	
N0	34	20 (58.8%)	14 (41.2%)		36	21 (58.3%)	15 (41.7%)		70	41 (58.6%)	29 (41.4%)	
TNM stage				0.011				0.013				< 0.001
III	82	29 (35.4%)	53 (64.6%)		96	35 (36.5%)	61 (63.5%)		178	64 (36.0%)	114 (64.0%)	
I + II	56	32 (57.1%)	24 (42.9%)		54	31 (57.4%)	23 (42.6%)		110	63 (57.3%)	47 (42.7%)

**Table 2:** Univariate and Multivariate COX regression analysis of prognostic factors associated with OS in training cohort

Variables	Univariate analysis		Multivariate analysis	
	HR (95% CI)	*p* value	HR (95% CI)	*p* value
Gender (female vs. male)	0.990 (0.643–1.525)	0.964	–	–
Age (years) (≥ 60 vs. < 60)	1.348 (0.887–2.049)	0.162	–	–
Tumor size (cm) (< 4 vs. ≥ 4)	0.711 (0.470–1.075)	0.106	–	–
Differentiation status (well + moderate vs. poor and undifferentiated)	0.738 (0.462–1.178)	0.203	–	–
Lauren type (diffuse type vs. intestinal type)	1.303 (0.850–1.996)	0.224	–	–
Depth of invasion (T1 + T2 vs. T3 + T4)	0.426 (0.261–0.695)	0.001	0.751 (0.445–1.268)	0.285
Lymph node metastasis (N0 vs. N1 + N2 + N3)	0.238 (0.119–0.477)	< 0.0001	0.961 (0.355–2.599)	0.937
TNM stage (I + II vs. III)	0.186 (0.107–0.325)	< 0.0001	0.211 (0.095–0.467)	< 0.001
CPNE3 (high vs. low)	1.751 (1.138–2.693)	0.011	1.555 (1.003–2.412)	0.049

In the validation cohort, patients with high CPNE3 expression showed substantially shorter OS (MST = 25 months) than those with low CPNE3 expression (MST = 51 months, *p* = 0.0036) (Fig. 8d). The findings of multivariate and univariate Cox regression analyses demonstrated that the expression of CPNE3 was a distinct predictive factor for GC (HR = 1.540, 95% CI 1.006–2.358; *p* = 0.047) (Table 3). According to the Kaplan–Meier survival analysis, high CPNE3 expression levels were associated with shorter OS (*p* < 0.0001) in the training and validation cohorts (Fig. 8e). Notably, the outcomes of the univariate and multivariate Cox analyses supported the notion that CPNE3 is a standalone prognostic factor for GC (HR = 1.525, 95% CI 1.124–2.071, *p* = 0.007) (Table 4). Thus, we conclude that increased CPNE3 expression is associated with a worse prognosis and may function as a standalone prognostic predictor in GC.

**Table 3:** Univariate and multivariate COX regression analysis of prognostic factors associated with OS in validation cohort

Variables	Univariate analysis		Multivariate analysis	
	HR (95% CI)	*p* value	HR (95% CI)	*p* value
Gender (female vs. male)	0.776 (0.518–1.162)	0.218	–	–
Age (years) (≥ 60 vs. < 60)	0.969 (0.657–1.429)	0.874	–	–
Tumor size (cm) (< 4 vs. ≥ 4)	0.889 (0.601–1.314)	0.555	–	–
Differentiation status (well + moderate vs. poor and undifferentiated)	0.750 (0.501–1.123)	0.162	–	–
Lauren type (diffuse type vs. intestinal type)	0.788 (0.534–1.162)	0.230	–	–
Depth of invasion (T1 + T2 vs. T3 + T4)	0.571 (0.373–0.875)	0.010	0.967 (0.596–1.571)	0.893
Lymph node metastasis (N0 vs. N1 + N2 + N3)	0.346 (0.192–0.621)	< 0.001	0.958 (0.376–2.440)	0.929
TNM stage (I + II vs. III)	0.321 (0.195–0.528)	< 0.0001	0.361 (0.158–0.826)	0.016
CPNE3 (high vs. low)	1.810 (1.204–2.720)	0.004	1.540 (1.006–2.358)	0.047

**Table 4:** Univariate and Multivariate COX regression analysis of prognostic factors associated with OS in training + validation cohort

Variables	Univariate analysis		Multivariate analysis	
	HR (95% CI)	*p* value	HR (95% CI)	*p* value
Gender (female vs. male)	0.873 (0.650–1.173)	0.368	–	–
Age (years) (≥ 60 vs. < 60)	1.135 (0.854–1.508)	0.383	–	–
Tumor size (cm) (< 4 vs. ≥ 4)	0.798 (0.601–1.060)	0.119	–	–
Differentiation status (well + moderate vs. poor and undifferentiated)	0.757 (0.559–1.025)	0.072	–	–
Lauren type (diffuse type vs. intestinal type)	0.995 (0.748–1.324)	0.973	–	–
Depth of invasion (T1 + T2 vs. T3 + T4)	0.503 (0.365–0.692)	< 0.0001	0.887 (0.625–1.260)	0.503
Lymph node metastasis (N0 vs. N1 + N2 + N3)	0.292 (0.186–0.456)	< 0.0001	0.973 (0.495–1.914)	0.938
TNM stage (I + II vs. III)	0.247 (0.171–0.359)	< 0.0001	0.277 (0.157–0.489)	< 0.0001
CPNE3 (high vs. low)	1.783 (1.326–2.397)	< 0.001	1.525 (1.124–2.071)	0.007

Kaplan–Meier survival analysis of data obtained from the GEO database demonstrated that high CPNE3 levels were associated with poorer OS (*p* = 0.0032) and FP (*p* = 0.012) in patients with GC exhibiting high *YAP1* mRNA expression, whereas OS and FP in patients with GC with low YAP1 levels were not significantly different between different CPNE3 levels (Supplemental Fig. S4F–I). Consequently, CPNE3 and YAP1 have been hypothesized to work together to worsen the prognosis of patients with GC. Subsequently, we verified the relationship between CPNE3 and the Hippo-YAP1 pathway using clinical patient samples. The expression of CPNE3, YAP1, CYR61, and RAD51 proteins in tumor tissue samples from 100 randomly selected patients with GC was examined using continuous-section IHC labeling. IHC of continuous sections obtained from the same patient's cancerous and non-cancerous tissues revealed that CPNE3, YAP1, CYR61, and RAD51 were significantly positive in the tumor tissue and negative in the non-cancerous tissue (Fig. 8f). Further survival analysis showed (Fig. 8g) that patients exhibiting high levels of both CPNE3 and YAP1 expression demonstrated comparatively shorter OS than those with high CPNE3 expression alone, while patients with concurrently low CPNE3 and YAP1 expression had the best prognosis.

## Discussion

To the best of our knowledge, this is the first study to demonstrate *CPNE3* is a direct target of the YAP1/TEADs transcription factor complex. CPNE3 maintains YAP1 stability by competitively binding YAP1 with β-TRCP, thereby inhibiting the ubiquitination of the YAP1 protein and eventually creating a positive feedback loop to encourage the malignant growth of GC cells.

Literature describing the regulatory network of downstream target genes of YAP1 in GC is scarce [28, 29]. Therefore, after suppressing the expression of *YAP1* in GC cell lines, we performed mRNA sequencing to identify possible target genes downstream of YAP1 and found that *CPNE3* was a new target gene downstream of *YAP1*. We performed RT-qPCR and WB to confirm these findings, which suggested that *CPNE3* is a direct target gene regulated by the YAP1/TEADs transcription factor complex. Additionally, using luciferase reporter gene and ChIP assays, *CPNE3* was identified as a directly regulated target gene downstream of YAP1. The molecular mechanism underlying the high expression of CPNE3 remains unknown, but it has been suggested that miR-133b directly regulates target *CPNE3* mRNA levels [30]. In this study, we elucidated a novel transcriptional regulatory mechanism for CPNE3.

To clarify the biological function of CPNE3 in GC, we downregulated the expression of CPNE3 in GC cells and found that the proliferation, invasion, and chemoresistance of GC cells were inhibited, whereas the overexpression of CPNE3 had the opposite biological effects. We used Crisp-Cas9 gene editing to construct a BGC-823 cell line with a stable knockdown of CPNE3, CDX, and PDX, which showed that targeting CPNE3 significantly inhibited tumor growth. Understanding the physiological functions of CPNE3 would be beneficial for identifying adverse reactions. CPNE3 has been reported to have the following physiological functions: it protects the heart against ischemia/reperfusion injury [31], modifies the relationship between anxiety and working memory [32], regulates insulin secretion and glucose uptake in pancreatic cells [33]; and low CPNE3 expression is associated with an increased risk of acute myocardial infarction [34]. We performed cellular assays to determine the function of CPNE3 in gastric epithelial cells after downregulation of CPNE3 expression in GES-1 cells. The results showed that downregulation of CPNE3 had slight effect on the proliferation, migration, invasion, and chemosensitivity of GES-1 cells. As CPNE3 has fewer toxic effects on normal tissues, *CPNE3* may serve as a promising drug target.

The available knowledge regarding the CPNE3 downstream pathway is limited. We found that CPNE3 modulates the YAP1 pathway without significantly affecting the expression of LATS1/2, an upstream regulator of YAP1, indicating that LATS kinase is not necessary for CPNE3 to regulate YAP1 [35]. Moreover, CPNE3 overexpression or deletion did not significantly change the expression of *YAP1* mRNA, indicating that CPNE3 controls YAP1 function in GC cells by influencing post-transcriptional or protein degradation levels [36, 37]. Here, we demonstrated that CPNE3 inhibits E3 ubiquitin ligase recruitment by competitively binding YAP1 to β-TRCP, prolonging the protein half-life of YAP1, and promoting YAP1 entry into the nucleus to activate the transcription of target genes downstream of the Hippo pathway, so forming a positive feedback loop [27, 38]. The recruitment of β-TRCP ubiquitin ligase to the C-terminal region of YAP1 facilitates its ubiquitination and degradation [39]. We speculate that CPNE3 binds to the C-terminal region of YAP1 and prevents its ubiquitination by TRCP ligase, leading to the stabilization and activation of YAP1.

However, the clinical significance of CPNE3 in GC has not been reported. In this study, the expression of CPNE3 in GC tissues was significantly elevated according to bioinformatics analysis and IHC, and positively correlated with the degree of malignancy of GC. The discovery and validation groups showed that patients with high CPNE3 expression had a poor prognosis, whereas patients with high YAP1 and CPNE3 expression had the worst clinical prognosis. These findings indicated that YAP1 and CPNE3 jointly drive malignant progression and chemotherapy resistance in GC. There is a relationship between CPNE3, some chemotherapy drugs, and targeted drug resistance [40]; therefore, the choice of chemotherapy regimen for patients with high CPNE3 and YAP1 expression may require some adjustment.

Three approaches for Hippo-YAP1-targeted therapy are available. The first approach involves the use of drugs, such as a super-TDU peptide, which can inhibit the YAP/TEAD interaction, thus decreasing the expression of YAP1 and its target genes [41]. The second approach involves the inhibition of YAP/TEAD activity, such as blocking oncogenic YAP/TAZ signaling using a variant pan-TEAD inhibitor and overcoming KRAS G12C inhibitor resistance [42]. The third approach involves therapies that target the upstream or downstream target genes of YAP1 [43]. However, these modalities have not shown significant clinical efficacy, and currently registered drugs targeting the Hippo-YAP1 signaling pathway are extremely limited. According to this study, the use of CRISPR-Cas9 to silence CPNE3 may open new possibilities for treating GC with YAP1 activation.

In conclusion, by validating its systematic biological function, molecular mechanism, and clinical applications, we have demonstrated that CPNE3 promotes GC cell proliferation, metastasis, and chemoresistance by acting as an oncogene in a YAP1-partially-dependent manner. Moreover, CPNE3 competitively binds YAP1 to β-TRCP, thereby inhibiting subsequent ubiquitination of the YAP1 protein and maintaining YAP1 stability, thus creating a malignant positive feedback loop. These findings provide a new approach for targeted therapy and identification of prognostic biomarkers for GC.

### Supplementary Information

Below is the link to the electronic supplementary material.

Supplementary file 1 (DOCX 1582 KB)

Supplementary file 2 (XLSX 15 KB)

## Data Availability

The data generated in this study are available within the article and its supplementary data files, and requests for additional raw data should be sent to the corresponding author.

## References

[1] Chen W, Zheng R, Baade PD (2016). Cancer statistics in China, 2015. CA Cancer J Clin.

[2] Al-Batran SE, Homann N, Pauligk C (2017). Effect of neoadjuvant chemotherapy followed by surgical resection on survival in patients with limited metastatic gastric or gastroesophageal junction cancer: the AIO-FLOT3 trial. JAMA Oncol.

[3] Sasako M, Sakuramoto S, Katai H (2011). Five-year outcomes of a randomized phase III trial comparing adjuvant chemotherapy with S-1 versus surgery alone in stage II or III gastric cancer. J Clin Oncol.

[4] Siegel RL, Miller KD, Wagle NS (2023). Cancer statistics, 2023. CA Cancer J Clin.

[5] Choi W, Kim J, Park J (2018). YAP/TAZ initiates gastric tumorigenesis via upregulation of MYC. Cancer Res.

[6] Kang W, Tong JH, Chan AW (2011). Yes-associated protein 1 exhibits oncogenic property in gastric cancer and its nuclear accumulation associates with poor prognosis. Clin Cancer Res.

[7] Liu H, Liu Y, Bian Z (2018). Circular RNA YAP1 inhibits the proliferation and invasion of gastric cancer cells by regulating the miR-367-5p/p27 (Kip1) axis. Mol Cancer.

[8] Song M, Cheong JH, Kim H (2012). Nuclear expression of yes-associated protein 1 correlates with poor prognosis in intestinal type gastric cancer. Anticancer Res.

[9] Qiao Y, Lin SJ, Chen Y (2016). RUNX3 is a novel negative regulator of oncogenic TEAD-YAP complex in gastric cancer. Oncogene.

[10] Zhao J, Han Z, Xu C (2023). Separation and single-cell analysis for free gastric cancer cells in ascites and peritoneal lavages based on microfluidic chips. EBioMedicine.

[11] Liu Z, Li J, Ding Y (2022). USP49 mediates tumor progression and poor prognosis through a YAP1-dependent feedback loop in gastric cancer. Oncogene.

[12] Bum-Erdene K, Zhou D, Gonzalez-Gutierrez G (2019). Small-molecule covalent modification of conserved cysteine leads to allosteric inhibition of the TEAD⋅Yap protein–protein interaction. Cell Chem Biol.

[13] Giraud J, Molina-Castro S, Seeneevassen L (2020). Verteporfin targeting YAP1/TAZ-TEAD transcriptional activity inhibits the tumorigenic properties of gastric cancer stem cells. Int J Cancer.

[14] Pobbati AV, Hong W (2020). A combat with the YAP/TAZ-TEAD oncoproteins for cancer therapy. Theranostics.

[15] Caudell EG, Caudell JJ, Tang CH (2000). Characterization of human copine III as a phosphoprotein with associated kinase activity. Biochemistry.

[16] Cowland JB, Carter D, Bjerregaard MD (2003). Tissue expression of copines and isolation of copines I and III from the cytosol of human neutrophils. J Leukoc Biol.

[17] Choi HY, Park N, Na JB (2016). Direct binding of Copine3 with Jab1 activates downstream ErbB2 signaling and motility in SKBr 3 breast cancer cells. Oncol Rep.

[18] Sun B, Li Y, Zhou Y (2019). Circulating exosomal CPNE3 as a diagnostic and prognostic biomarker for colorectal cancer. J Cell Physiol.

[19] Lin H, Zhang X, Liao L (2018). CPNE3 promotes migration and invasion in non-small cell lung cancer by interacting with RACK1 via FAK signaling activation. J Cancer.

[20] Yao Y, Liu Z, Huang S (2022). The E3 ubiquitin ligase, FBXW5, promotes the migration and invasion of gastric cancer through the dysregulation of the Hippo pathway. Cell Death Discov.

[21] Li L, Zhao J, Huang S (2018). MiR-93-5p promotes gastric cancer-cell progression via inactivation of the Hippo signaling pathway. Gene.

[22] Huang S, Cao Y, Guo H (2020). Up-regulated acylglycerol kinase (AGK) expression associates with gastric cancer progression through the formation of a novel YAP1-AGK-positive loop. J Cell Mol Med.

[23] Liu Z, Huang S, Cao Y (2018). YAP1 inhibits circRNA-000425 expression and thus promotes oncogenic activities of miR-17 and miR-106. Biochem Biophys Res Commun.

[24] Elaimy AL, Amante JJ, Zhu LJ (2019). The VEGF receptor neuropilin 2 promotes homologous recombination by stimulating YAP/TAZ-mediated Rad51 expression. Proc Natl Acad Sci USA.

[25] Hsu YL, Hung JY, Chou SH (2015). Angiomotin decreases lung cancer progression by sequestering oncogenic YAP/TAZ and decreasing Cyr61 expression. Oncogene.

[26] Yao F, Zhou Z, Kim J (2018). SKP2- and OTUD1-regulated non-proteolytic ubiquitination of YAP promotes YAP nuclear localization and activity. Nat Commun.

[27] Zhao B, Li L, Tumaneng K (2010). A coordinated phosphorylation by Lats and CK1 regulates YAP stability through SCF(beta-TRCP). Genes Dev.

[28] Lin MT, Zuon CY, Chang CC (2005). Cyr61 induces gastric cancer cell motility/invasion via activation of the integrin/nuclear factor-kappaB/cyclooxygenase-2 signaling pathway. Clin Cancer Res.

[29] Liu M, Yao B, Gui T (2020). PRMT5-dependent transcriptional repression of c-Myc target genes promotes gastric cancer progression. Theranostics.

[30] Mo W, Zhang J, Li X (2013). Identification of novel AR-targeted microRNAs mediating androgen signalling through critical pathways to regulate cell viability in prostate cancer. PLoS ONE.

[31] Zhang X, Han X, Zhang Y (2022). CPNE3 interaction with RACK1 protects against myocardial ischemia/reperfusion injury. Exp Ther Med.

[32] Chen C, Wang Z, Chen C (2021). CPNE3 moderates the association between anxiety and working memory. Sci Rep.

[33] El-Huneidi W, Anjum S, Mohammed AK (2021). Copine 3 “CPNE3” is a novel regulator for insulin secretion and glucose uptake in pancreatic beta-cells. Sci Rep.

[34] Tan B, Liu L, Yang Y (2019). Low CPNE3 expression is associated with risk of acute myocardial infarction: a feasible genetic marker of acute myocardial infarction in patients with stable coronary artery disease. Cardiol J.

[35] Seo J, Kim MH, Hong H (2019). MK5 regulates YAP stability and is a molecular target in YAP-driven cancers. Cancer Res.

[36] Yuan B, Liu J, Shi A (2023). HERC3 promotes YAP/TAZ stability and tumorigenesis independently of its ubiquitin ligase activity. Embo j.

[37] Zhu H, Yan F, Yuan T (2020). USP10 promotes proliferation of hepatocellular carcinoma by deubiquitinating and stabilizing YAP/TAZ. Cancer Res.

[38] Liu J, Bai W, Zhou T (2023). SDCBP promotes pancreatic cancer progression by preventing YAP1 from β-TrCP-mediated proteasomal degradation. Gut.

[39] Shanzer M, Adler J, Ricardo-Lax I (2017). The nonreceptor tyrosine kinase c-Src attenuates SCF(beta-TrCP) E3-ligase activity abrogating Taz proteasomal degradation. Proc Natl Acad Sci USA.

[40] Chen Z, Jiang Z, Zhang W (2018). Silencing the expression of copine-III enhances the sensitivity of hepatocellular carcinoma cells to the molecular targeted agent sorafenib. Cancer Manag Res.

[41] Jiao S, Wang H, Shi Z (2014). A peptide mimicking VGLL4 function acts as a YAP antagonist therapy against gastric cancer. Cancer Cell.

[42] Hagenbeek TJ, Zbieg JR, Hafner M (2023). An allosteric pan-TEAD inhibitor blocks oncogenic YAP/TAZ signaling and overcomes KRAS G12C inhibitor resistance. Nat Cancer.

[43] Haemmerle M, Taylor ML, Gutschner T (2017). Platelets reduce anoikis and promote metastasis by activating YAP1 signaling. Nat Commun.

