# Supplementary material for: YAP1-CPNE3 positive feedback pathway promotes gastric cancer cell progression
Source: Cell Mol Life Sci. 2024 Mar 17;81(1):143. doi: 10.1007/s00018-024-05178-3 (PMC10944813; doi:10.1007/s00018-024-05178-3)
Supplement: Supplementary file 1 — (DOCX 1582 KB) [file 18_2024_5178_MOESM1_ESM.docx]

**YAP1-CPNE3 positive feedback pathway promotes gastric cancer cell progression**

Xuan Li^1#^, Hongguang Zhong^1#^, Qianqian Shi^1#^, Ruiwen Ruan^1#^, Chunye Huang^1^, Qin Wen^1^, Shaocheng Zeng^1^, Yang Xia^1^, Qinru Zeng^1^, Jianping Xiong^1,2^, Shanshan Wang^3^*, Jun Chen^1^*, Wan Lei^3^*, Jun Deng^1,2,4^*

^1^Department of Oncology, The First Affiliated Hospital, Jiangxi Medical College, Nanchang University, Nanchang, Jiangxi, China

^2^Jiangxi Key Laboratory for Individual Cancer Therapy, Nanchang Jiangxi, China

^3^Department of Pathology, The First Affiliated Hospital, Jiangxi Medical College, Nanchang University, Nanchang, Jiangxi, China

^4^Postdoctoral Innovation Practice Base，The First Affiliated Hospital of Nanchang University, Nanchang, 330006, People's Republic of China

Xuan Li, 17 Yongwaizheng Street, Nanchang City, Jiangxi Province. Email: lxuan2021@126.com

Hongguang Zhong, 17 Yongwaizheng Street, Nanchang City, Jiangxi Province. Email: zhg9607@126.com

Qianqian Shi, 17 Yongwaizheng Street, Nanchang City, Jiangxi Province. Email: 1815464755@qq.com

Ruiwen Ruan, 17 Yongwaizheng Street, Nanchang City, Jiangxi Province. Email: 564370264@qq.com

Chunye Huang, 17 Yongwaizheng Street, Nanchang City, Jiangxi Province. Email: 824103249@qq.com

Qin Wen, 17 Yongwaizheng Street, Nanchang City, Jiangxi Province. Email: 3145519090@qq.com

Shaocheng Zeng, 17 Yongwaizheng Street, Nanchang City, Jiangxi Province. Email: 1689897425@qq.com

Yang Xia, 17 Yongwaizheng Street, Nanchang City, Jiangxi Province. Email: 1614322487@qq.com

Qinru Zeng, 17 Yongwaizheng Street, Nanchang City, Jiangxi Province. Email: 2825805355@qq.com

Jianping Xiong, 17 Yongwaizheng Street, Nanchang City, Jiangxi Province. Email: Jpxiong0630@outlook.com

***Corresponding authors:**

Jun Deng, 17 Yongwaizheng Street, Nanchang City, Jiangxi Province. Phone: 0791-88692748; email: dengjun19871106@ncu.edu.cn

Wan Lei, 17 Yongwaizheng Street, Nanchang City, Jiangxi Province. Phone:

0791-88692748; email: 522569951@qq.com

Jun Chen, 17 Yongwaizheng Street, Nanchang City, Jiangxi Province. Phone:

0791-88692748; email: chyf1011@163.com

Shanshan Wang, 17 Yongwaizheng Street, Nanchang City, Jiangxi Province. Phone: 0791-88692748; email: swansea33@163.com

**Competing interests:** The authors declare no competing financial interests.

**Keywords: Gastric cancer, CPNE3, YAP1, TEADs**

**The Supplementary Figure legends were described in this PDF files:**

**Supplementary Figure 1**

(A) *CPNE3* expression differences between tumor and normal tissues in pan-cancer from the GEPIA database. (B, C) Correlation of CPNE3 with YAP1, TEAD1, TEAD4 using expression data from single cell sequencing. (D) The predicted complexes' interfaces were shown. (E) Analysis and visualization of the interface area and free energy of the predicted complexes. It follows that proteins will attach to one another more readily if the contact area is greater. When the free energy is negative, a protein may bind in a stable manner.

Supplementary Figure 2

(A) The protein levels of CPNE3, YAP1, CYR61 in HGC-27 and AGS cells transfected with siNC, siYAP1-#1 or siYAP1-#2 were detected by WB assay and the results were quantified. (B) After gradient overexpression of Flag-YAP1, the protein levels of YAP1, CPNE3, and CYR61 were detected by WB and analyzed statistically. (C) In AGS cells stably expressing YAP1-WT, YAP-5SA, or YAP-S94A, the expression of CPNE3 and YAP1 was detected and quantitatively analyzed on WB with the corresponding antibodies. (D) The YAP1-WT plasmid was introduced into TEADs-deficient BGC-823 cells and the results were analyzed by WB and quantified. (E) Using siRNA, the expression of CPNE3 was down-regulated in BGC-823 and MKN-28 cells, and the expression of CPNE3, LATS1, LATS2, YAP1, CYR61, RAD51 proteins was detected and quantified by WB. (F) Up-regulation of CPNE3 expression by transfection of HA-CPNE3 plasmid in AGS and HGC-27 cells. The expression of CPNE3, LATS1, LATS2, YAP1, CYR61, and RAD51 proteins was examined by WB and quantified. (G) BGC-823 and MKN-28 cells were treated with the ShCPNE3-#2 plasmid to downregulate the expression of CPNE3 and the Flag-YAP1 plasmid to concurrently increase the expression of YAP1, and the protein level of CPNE3, YAP1, and CYR61 was detected and quantified by WB. (H) CPNE3 expression was down-regulated in MKN-45 cells lacking YAP1 expression, and the protein level of CPNE3 was detected and quantified by WB assay. (I) ShYAP1-#1 plasmid-mediated stable *YAP1*knockdown or HA-CPNE3 plasmid-mediated simultaneous overexpression of CPNE3 in AGS and HGC-27 cells, and the protein level of CPNE3, YAP1, and CYR61 was detected and quantified by WB. (J)The protein level of CPNE3 was detected and quantified by WB assay after transfecting GES-1 cells with NC, siCPNE3-#1, or siCPNE3-#2. Three independent biological experiments were conducted, which consistently yielded similar results. Statistical significance is indicated by *p <0.05 and **p <0.01. Scale bar: 200 μm.

**Supplementary Figure 2**

(A) The protein level of CPNE3 was detected by WB after transfecting GES-1 cells with NC, siCPNE3-#1, or siCPNE3-#2. (B–E) After down-regulation of CPNE3 with siRNA, the proliferation, migration and invasion ability of GES-1 cells were detected by CCK-8 and transwell assays, respectively, and drug sensitivity assay was used to detect drug resistance in normal gastric epithelial cell. Three independent biological experiments were performed, and yielded similar results in each independent biological experiment, and statistical significance is denoted by *p <0.05 and **p <0.01. Scale bar: 200 μm.

**Supplementary Figure 4**

(A) *CPNE3* expression was higher in GC tissues (n = 415) than in normal tissues (n = 34) in the Ualcan database. (B) High *CPNE3* mRNA levels were associated with TNM staging in GC tissues. (C–E) Analysis of the Kaplan-Meier plotter database revealed that the overall survival of GC patients with high CPNE3 expression (n = 498) was significantly shorter (p = 0.0058, hazard ratio [HR] = 1.28, 95 % confidence interval [CI]: 1.07-1.52) than that of patients with low CPNE3 expression (n = 377). Similarly, the PPS of patients with high CPNE3 expression (n = 189) was significantly shorter (p = 2.6e-08, HR = 1.89, 95 % CI: 1.49-3.2) than that of patients with low *CPNE3* expression (n = 309), the FP of patients with high CPNE3 expression (n = 332) was significantly shorter (p = 0.036, HR = 1.24, 95 % CI: 1.01 − 1.52) than that of patients with low CPNE3 expression (n = 308). (*p <0.05, **p <0.01). (F–I) Kaplan-Meier survival analysis of GC patients was carried out based on the GEO database. Patients were grouped according to CPNE3 (202118_s_at) expression and stratified according to *YAP1* (213342_at) mRNA expression, and the results showed that high *CPNE3* mRNA level only resulted in poorer OS (p = 0.0032) and FP (p = 0.012) in GC patients with high YAP1 level.

**
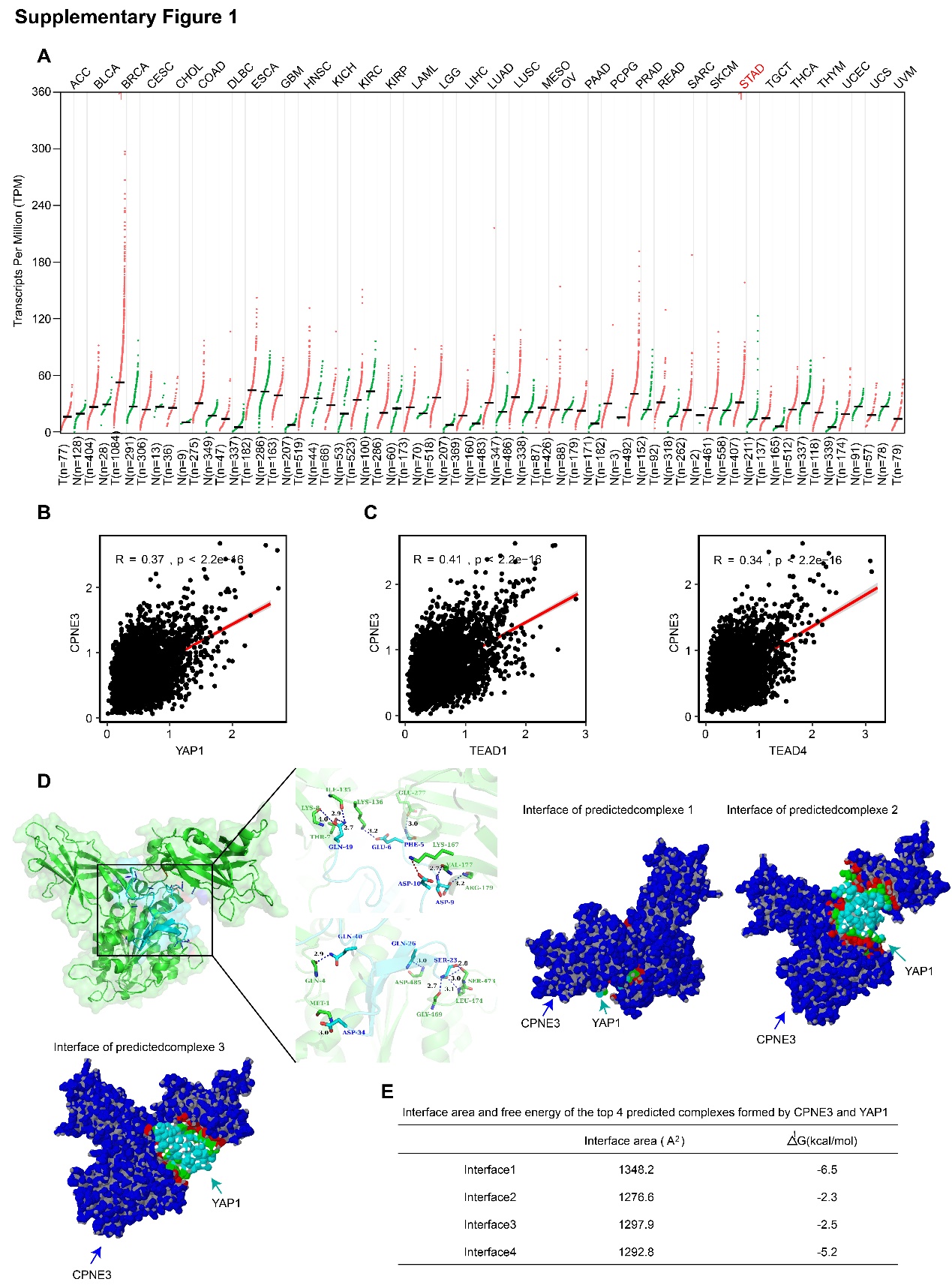
**

**
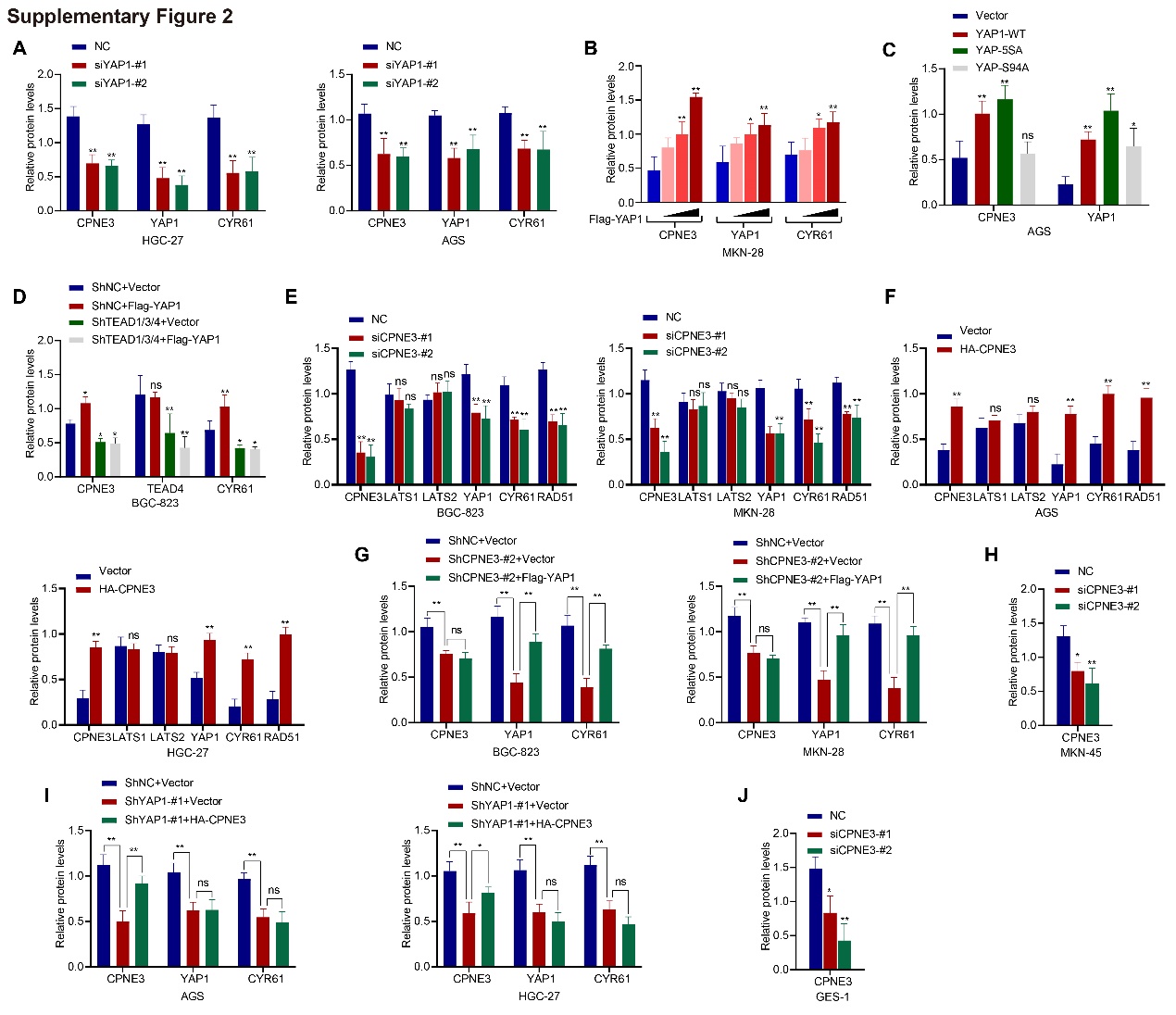
**


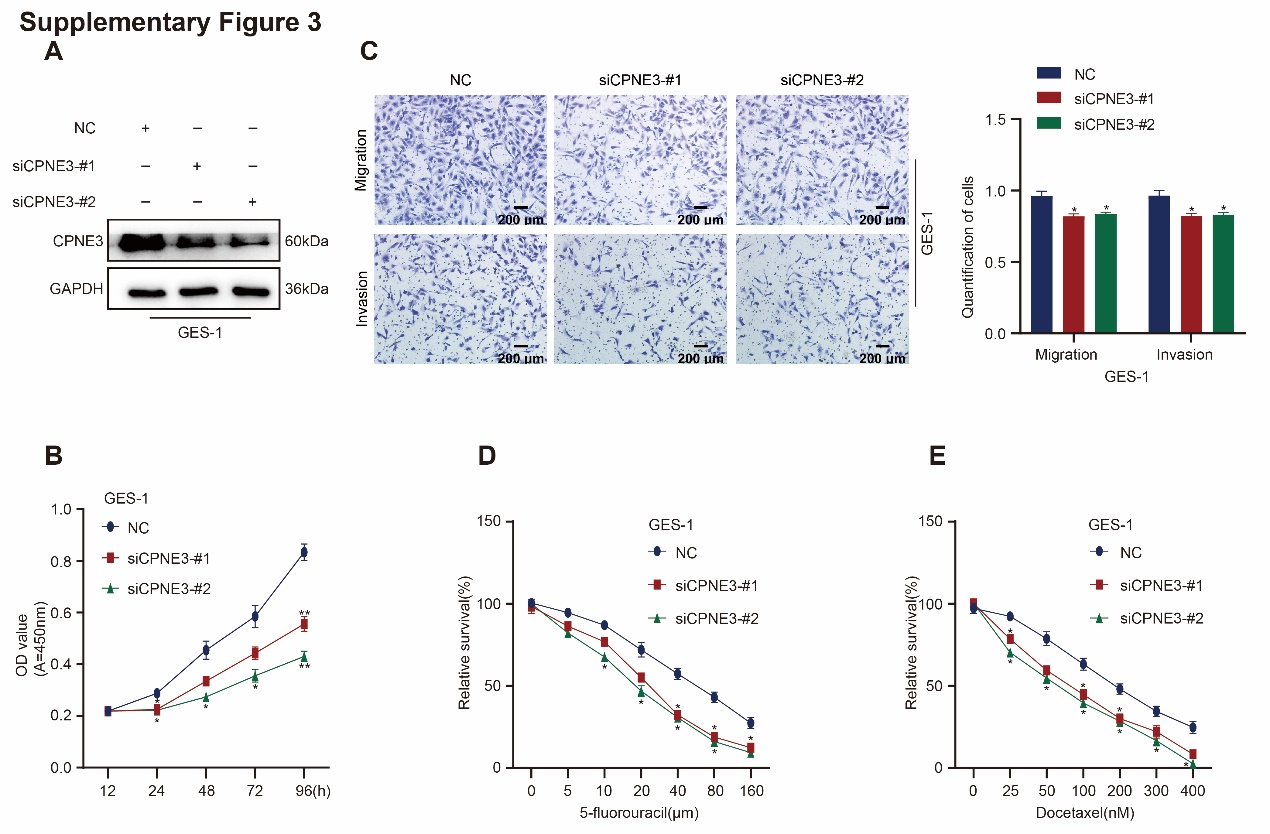


**
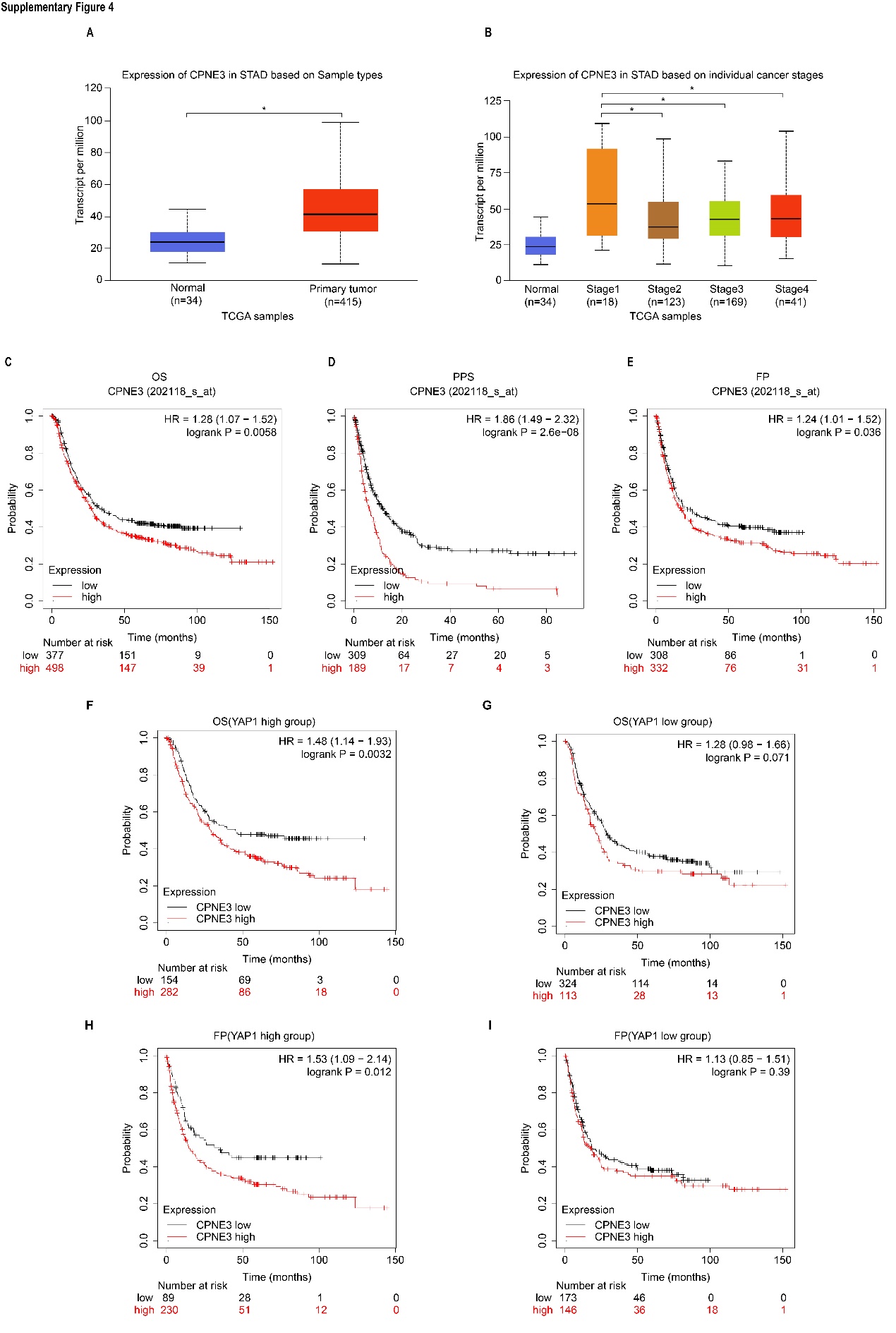
**

|  |
| --- |

| Supplementary Table 1. Eight pairs of information for patients with fresh gastric cancer tissue. | | | | | | |
| --- | --- | --- | --- | --- | --- | --- |
| Gender | Age | Sample Information | Clinical diagnosis | Type of sample use | Sample Detailed Parameters | Sample collection time |
| Male | 71 | Patient's postoperative tissues | Malignant tumor of the stomach | Preparation of proteins | Tissue size: T:0.6*0.5cm N:1*1.5cm | November 7, 2022 |
| Male | 70 | Patient's postoperative tissues | Malignant tumor of the stomach | Preparation of proteins | Tissue size: T:1*0.5cm N:1*1cm | February 16, 2023 |
| Male | 68 | Patient's postoperative tissues | Malignant tumor of the stomach | Preparation of proteins | Tissue size: T:0.3*0.5cm N:1*0.8cm | March 7, 2023 |
| Male | 73 | Patient's postoperative tissues | Malignant tumor of the stomach | Preparation of proteins | Tissue size: T:1*0.5cm N:1*1cm | March 8, 2023 |
| Male | 64 | Patient's postoperative tissues | Malignant tumor of the stomach | Preparation of proteins | Tissue size: T:0.5*0.4cm N:2*1.5cm | March 13, 2023 |
| Female | 74 | Patient's postoperative tissues | Malignant tumor of the stomach | Preparation of proteins | Tissue size: T:0.2*0.2cm N:1*1.5cm | March 14, 2023 |
| Female | 60 | Patient's postoperative tissues | Malignant tumor of the stomach | Preparation of proteins | Tissue size: T:0.8*1cm N:2*2cm | March 28, 2023 |
| Male | 67 | Patient's postoperative tissues | Malignant tumor of the stomach | Preparation of proteins | Tissue size: T:0.5*0.5cm N:1.5*1.5cm | March 29, 2023 |

|  |
| --- |

| Supplementary Table 2 | Primary antibodies used in this research | |
| --- | --- | --- |
| Primary antibody name | Source | ldentifier |
| CPNE3 | Proteintech | Cat#11186-1-AP |
| CPNE3 | abcam | Cat#ab236606 |
| YAP1 | Proteintech | Cat#66900-1-Ig |
| YAP1 | Cell Signaling Technology | Cat#D8H1X |
| RAD51 | Proteintech | Cat#11126-1-AP |
| CYR61 | Proteintech | Cat#26689-1-AP |
| Flag-Tag | Proteintech | Cat#66008-4-Ig |
| Flag-Tag | PTM BIO | Cat#6075 |
| His-Tag | Proteintech | Cat#66005-1-Ig |
| Myc-Tag | Proteintech | Cat#10828-1-AP |
| HA-Tag | Proteintech | Cat#66006-2-Ig |
| HA-Tag | PTM BIO | Cat#5177 |
| ubiquitin | Proteintech | Cat#10201-2-AP |
| LATS1 | Cell Signaling Technology | Cat#3477 |
| LATS2 | abcam | Cat#ab110780 |
| TEAD4 | Proteintech | Cat#12418-1-AP |
| GAPDH | Proteintech | Cat#10494-1-AP |
| β-actin | Proteintech | Cat#66009-1-Ig |

| Supplementary Table 3 | The primer sequences used in this research |
| --- | --- |
| Gene | Sequence |
| GAPDH | F 5’-GGAAGCTTGTCATCAATGGAAATC-3’  R 5’-TGATGACCCTTTTGGCTCCC-3’ |
| Bmp4 | F 5’-AGGAGGAAGAGCAGATCCACAG-3’  R 5’-GGATGTTCTCCAGATGTTCTTCGT-3’ |
| CXCR4 | F 5’-GCCTTATCCTGCCTGGTATTGT-3’  R 5’-AGGATGACTGTGGTCTTGAGGG-3’ |
| CCSAP | F 5’-GGAAACAGGAAAGCGGTCAA-3’  R 5’-ATTCTGTCATCCACGGGTTCTC-3’ |
| KLHL23 | F 5’-GAAGAGGCTGAGTTCTATGATCCTT-3’  R 5’-ATGTGGACTGGAGGTGATGAGG-3’ |
| MAGT1 | F 5’-GCAGTCAAGCCCAGTTTGTAGC-3’  R 5’-CAGCCACACACATTATCTTTCGC-3’ |
| MALT1 | F 5’-GTGTTGCTGTTGGAAGCCCTAT-3’  R 5’-TCCACTGCCTCATCTGTTCTTC-3’ |
| NPTX1 | F 5’-ATGTATGCCAAGGTGAAGAAGAGC-3’  R 5’-GTGGTGCCACTTGCCATCATT-3’ |
| PCDH20 | F 5’-AGTACGTGACCCTAGACAACCG-3’  R 5’-CAAGCACATCCAGCAGCAAA-3’ |
| CPNE3 | F 5’ -ATTTGGGGTTTATGACATCGACA-3’  R 5’-GCTGAAATCGTAATGCTCCCTT-3’ |
| CYR61 | F 5’-CAGGACTGTGAAGATGCGGT-3’  R 5’-GCCTGTAGAAGGGAAACGCT-3’ |
| FSTL1 | F 5’- GCCATGACCTGTGACGGAAA -3'  R 5’- CAGCGCTGAAGTGGAGAAGA -3' |
| YAP1 | F 5'-TCGTTTTGCCATGAACCAGA-3'  R 5'-GGCTGCTTCACTGGAGCACT-3' |
| RAD51 | F 5'-CTCGCTGATGAGTTTGGTGT-3'  R 5'-TTGCAGATTCTGGTTTCCC-3' |
| C-MYC | F 5'-GGTCTTCCCCTACCCTCTCAAC-3'  R 5'-GCTGTGAGGAGGTTTGCTGTG-3' |

| Supplementary Table 4 | Sequences of siRNAs/ShRNAs /LV-sgRNAs in this research | |
| --- | --- | --- |
| Product Name | Sequence | |
| SiCPNE3-#1 | Sense:  Antisense: | 5’-GGUUCAUCGGACAGAGGUUTT-3’  5’-AACCUCUGUCCGAUGAACCTT-3’ |
| SiCPNE3-#2 | Sense:  Antisense: | 5’-GCAAUGGAAUCCAAGGCAUTT-3’  5’-AUGCCUUGGAUUCCAUUGCTT-3’ |
| siYAP1-#1 | Sense:  Antisense: | 5’-CUGCCACCAAGCUAGAUAATT-3’  5’-UUAUCUAGCUUGGUGGCAGTT-3’ |
| siYAP1-#2 | Sense:  Antisense: | 5’-GGUGAUACUAUCAACCAAATT-3’  5’-UUUGGUUGAUAGUAUCACCTT-3’ |
| NC | Sense:  Antisense: | 5’-UUCUCCGAACGUGUCACGUT-3’  5’-ACGUGACACGUUCGGAGAATT-3’ |
| CPNE3 (Homo) cloning primer | Sense:  Antisense: | 5’-ACGGGCCCTCTAGACTCGAGCGCCACCATGGCTGCCCAGTGTGTCACAAAG-3’  5’-TCATAAGGGTACATGGATCCCTGCTTCTGTTGTTTCGTGGCTGGGTTC-3’ |
| ShCPNE3-#2 |  | 5’-GCAATGGAATCCAAGGCAT-3’ |
| ShNC |  | 5’-CGCTTCCGCGGCCCGTTCAA-3’ |
| LV-CPNE3-sgRNA (10823-1) |  | 5’-GTAATCAATAATAAATGTCT-3’ |
| LV-CPNE3-sgRNA (10824-1) |  | 5’-GGTTCAGAAATTGAAATTTG-3’ |
| LV-CPNE3-sgRNA (10825-1) |  | 5’-CATTGGGGCTGATGTAATGA-3’ |
| LV-NC |  | 5’-CGCTTCCGCGGCCCGTTCAA-3’ |

| Supplementary Table 5 | Sequences of Primers for ChIP assay in this research | |
| --- | --- | --- |
| CPNE3 promoter  TBS1 | Sense:  Antisense: | 5’-CCTGCTACCATTCCTGTGC-3’  5’-GGTGATGTGATAGAGGGACTTG-3’ |
| CPNE3 promoter  TBS2 | Sense:  Antisense: | 5’-AACACCAGCCTGAGCAAC-3’  5’-CAGATGATCCACCCACCTT-3’ |
| CPNE3 promoter  TBS3 | Sense:  Antisense: | 5’-TCCAACTCCTGGGCACAA-3’  5’-GCTTACTGAAAAGGCAAAACTG-3’ |

| Supplementary Table 6 | Databases used in this research |
| --- | --- |
| Database | URL |
| Swiss-model repository | https://swissmodel.expasy.org/ |
| RCSB PDB | https://www1.rcsb.org/ |
| ZDOCK | https://zdock.umassmed.edu/ |
| Proteinatlas | https://www.proteinatlas.org/ |
| Ualcan | http://ualcan.path.uab.edu/ |
| Kaplan-Meier plotter | http://kmplot.com/analysis/ |
| JASPAR | http://jaspar.genereg.net/ |
| GEPIA | http://gepia.cancer-pku.cn/ |

| Supplementary Table 7 | Explanation of abbreviations in this research |
| --- | --- |
| Abbreviations | Explanation |
| GC | Gastric Cancer |
| YAP1 | Yes-Associated Protein 1 |
| CPNE3 | copine 3 |
| LATS1/2 | Large tumor suppressors 1/2 |
| MST1/2 | mammalian Sterile 20-like kinase 1/2 |
| TEAD1/2/3/4 | Transcriptional enhanced associate domain transcription factors 1/2/3/4 |
| CYR61 | Cysteine-rich angiogenic inducer 61 |
| FSTL1 | Follistatin Like Protein 1 |
| CCSAP | centriole, cilia and spindle associated protein |
| KLHL23 | kelch like family member 23 |
| MAGT1 | magnesium transporter 1 |
| BMP4 | bone morphogenetic protein 4 |
| MALT1 | Mucosa-associated lymphoid tissue protein 1 |
| CXCR4 | C-X-C motif chemokine receptor 4 |
| NPTX1 | neuronal pentraxin 1 |
| PCDH20 | protocadherin 20 |
| WB | Western blotting |
| RT-qPCR | Real-time quantitative polymerase chain reaction |
| PBS | Phosphate buffer solution |
| CCK-8 | Cell Counting Kit-8 |
| NC | Negative control |
| siRNA | small interfering RNA |
| sgRNA | small guide RNA |
| OD | Optical density |
| CDX | Cell-line-derived xenograft |
| OS | Overall Survival |
| FBS | Fetal bovine serum |
| PPS | Post-progression survival |
| FP | First progression |
| IHC | Immunohistochemistry |
| ChIP | Chromatin Immunoprecipitation |
